# Intron retention is a robust marker of intertumoral heterogeneity in pancreatic ductal adenocarcinoma

**DOI:** 10.1038/s41525-020-00159-4

**Published:** 2020-12-11

**Authors:** Daniel J. Tan, Mithun Mitra, Alec M. Chiu, Hilary A. Coller

**Affiliations:** 1 Department of Molecular, Cell, and Developmental Biology, University of California, Los Angeles, CA USA; 2 Department of Biological Chemistry, David Geffen School of Medicine, University of California, Los Angeles, CA USA; 3 Bioinformatics Interdepartmental Program, University of California, Los Angeles, CA USA; 4 Molecular Biology Institute, University of California, Los Angeles, CA USA; 5 Present Address: Department of Biomedical Informatics, Harvard Medical School, Boston, MA USA

**Keywords:** Cancer genomics, Gene regulatory networks

## Abstract

Pancreatic ductal adenocarcinoma (PDAC) is an aggressive cancer with a 5-year survival rate of <8%. Unsupervised clustering of 76 PDAC patients based on intron retention (IR) events resulted in two clusters of tumors (IR-1 and IR-2). While gene expression-based clusters are not predictive of patient outcome in this cohort, the clusters we developed based on intron retention were associated with differences in progression-free interval. IR levels are lower and clinical outcome is worse in IR-1 compared with IR-2. Oncogenes were significantly enriched in the set of 262 differentially retained introns between the two IR clusters. Higher IR levels in IR-2 correlate with higher gene expression, consistent with detention of intron-containing transcripts in the nucleus in IR-2. Out of 258 genes encoding RNA-binding proteins (RBP) that were differentially expressed between IR-1 and IR-2, the motifs for seven RBPs were significantly enriched in the 262-intron set, and the expression of 25 RBPs were highly correlated with retention levels of 139 introns. Network analysis suggested that retention of introns in IR-2 could result from disruption of an RBP protein−protein interaction network previously linked to efficient intron removal. Finally, IR-based clusters developed for the majority of the 20 cancer types surveyed had two clusters with asymmetrical distributions of IR events like PDAC, with one cluster containing mostly intron loss events. Taken together, our findings suggest IR may be an important biomarker for subclassifying tumors.

## Introduction

Cancer of the pancreas is the fourth most common cause of cancer deaths for men and women in the United States^1^ with an estimated 57,600 new cases in 2020 and 47,050 deaths^1^. For all stages and types of pancreatic cancer combined, the 5-year relative survival rate is only 9%^1^. Pancreatic ductal adenocarcinoma (PDAC), cancer of the exocrine tissue of the pancreas, represents about 93% of all pancreatic cancers^1^. PDACs are usually not detected until they reach an advanced stage because symptoms do not usually appear until the disease is advanced^1^. Over half of PDAC patients (53%) are diagnosed at the distant stage, indicating the disease has spread from its original site, and for these patients, 5-year survival rate is only 3% ^2^.

The pancreas is composed of endocrine cells that secrete the hormones that regulate glucose metabolism (insulin and glucagon), and acinar cells that release digestive enzymes (zymogens) into pancreatic ducts. The pancreas also contains epithelial ductal cells that line the ducts^3^. Both ductal and acinar exocrine cells have been suggested as possible candidates for the cell of origin of PDACs^4^.

Four genes are frequently mutated in PDACs—KRAS (94% of tumors), TP53 (64%), SMAD4 (21%), and CDKN2A (17%)^5^. Despite these common oncogenic drivers, PDACs nevertheless exhibit a high degree of inter-tumor heterogeneity^4,6–8^ at genomic^9,10^, metabolic^11^, transcriptomic^12–17^, and histopathological^18^ levels. The three most commonly reported classification schemes for primary PDAC tumors are based on gene expression. Collisson et al. used microarray data to classify tumors (*n* = 85) into three subtypes: classical (epithelial), quasi-mesenchymal (QM), and exocrine-like^12^ based on the gene expression signatures. High-expression levels of epithelial gene markers were observed in the classical tumors; high levels of mesenchyme-associated genes were observed in the quasi-mesenchyme tumors and high expression of exocrine-associated genes was observed in the exocrine-like tumors. Survival outcomes were worse for patients with tumors in the QM group compared to patients in the classical group. Moffitt et al.^13^ classified PDACs (*n* = 145) into basal and classical subtypes based on microarray and RNA-seq data. Patients with tumors of the basal subtype (defined by expression of genes such as laminins and keratin) were found to have significantly poorer survival compared to tumors in the classical group, which overlapped with the classical group defined by Collisson et al. Finally, Bailey et al.^14^ discovered four subtypes of PDACs—squamous, immunogenic, pancreatic progenitor, and aberrantly differentiated exocrine (ADEX)—based on RNA-seq data from 266 tumors. These four subtypes were defined based on gene expression patterns of the transcriptional networks involved during the development and regeneration of pancreas. Patients with the squamous subtype had the worst survival compared to patients with the other three subtypes. PDAC tumors tend to have a dense stroma consisting of nontumor fibroblasts and immune cells, and consequently have low tumor purity^19^. The presence of these nontumor cells within the sample can be a confounding factor for transcriptome-based tumor analysis^15^. In a comprehensive study by The Cancer Genome Atlas (TCGA) network, 76 high-purity PDAC samples (ABSOLUTE purity ≥ 33%) were analyzed based on high-throughput genomic, transcriptomic, and proteomic data. The authors found that tumors with a basal classification (Moffitt et al.) overlapped with the tumors classified as squamous subtype (Bailey et al.), while the tumors designated with the classical subtype (Moffitt et al.) overlapped with the tumors assigned as pancreatic progenitor (Bailey et al.) and classical (Collisson et al.) subtypes. The data taken together suggest that high-purity PDACs can be divided into two groups based on gene expression: basal/squamous and classical/pancreatic progenitor.

While the analysis of gene expression in different pancreatic tumors has provided important insights into the subcategories of pancreatic cancer, understanding not just the total level of all isoforms of a gene, but also the levels of individual splicing isoforms may provide more insight into pancreatic cancer etiology and outcome^20,21^. Transcripts generated by alternative splicing (AS) can differ in their degradation, translation or localization^21^. AS can also produce transcripts from the same gene that, when translated, produce different proteins, potentially generating neoepitopes^22^, thereby vastly expanding the cellular regulatory landscape. Changes in AS have been associated with all the “hallmarks of cancer”^23^—replicative immortality, sustained proliferation, evasion of growth suppressors, and activation of invasion and metastasis^24^. RNA splicing factors are increasingly recognized as oncogenes and tumor suppressors^25^. AS can be dysregulated in cancer as a result of several factors, including mutations in splice sites, splicing factor mutations, changes in the expression levels of splicing factors, and altered post-translational modification of splicing factors^26–29^. A recent study argued that splicing and mutagenesis provide two independent paths to tumorigenesis^30^. Individual tumors were found to either contain a high level of mutations, or a large number of alternative splicing events, but not both^30^.

In a large-scale study of AS events in 32 cancer types (including pancreatic adenocarcinoma), the TCGA group found that tumor tissue contains 30% more AS events than normal tissue from the same site^31^. Changes in the use of polyadenylation sites leading to transcripts with different 3′ ends have been observed in PDACs with enrichment for alternative polyadenylation events in genes known to play a role in PDAC development^32^. Analyzing exon-specific microarray data from 28 PDACs and 6 normal pancreas tissues, Wang et al.^33^ found that exon skipping (14.3%), alternative first exon use (14%), and intron retention (8.4%) represented the three most prevalent categories of differentially spliced events comparing PDAC and normal samples. Wang et al. discovered two AS subtypes of PDAC that were not concordant with any of three gene expression-based subtypes identified by Collisson et al.^12^ These findings suggest that AS could be an independent predictor of inter-tumor heterogeneity in PDAC. Wang et al. did not, however, explore the separate contributions of different AS types, such as exon skipping or intron retention, to tumor-to tumor differences.

We sought to determine whether differences in patient outcome for tumors of the same type could be explained by differences among the tumors with respect to specific categories of AS. Focusing on PDAC, a tumor for which the existing subtypes have been developed based on gene expression data, we performed large-scale analysis of inter-tumor heterogeneity based on AS events. Our analysis captured the global landscape of AS events in 76 high-purity PDACs. Among the five AS types we investigated, intron retention proved to be a predictive biomarker that classifies PDAC patients into two clusters with divergent clinical outcomes.

## Results

### Robust clustering of PDAC patients based on intron retention events

We analyzed RNA-seq data from 76 high-purity PDAC samples collected from 76 PDAC patients^15^. Percent spliced in (PSI) values for all AS events for different AS types (exon skip (ES), intron retention (IR), alternative 5′/3′ splice site (A5/A3), multiple exon skip (ME), and mutually exclusive exons) were determined using Spladder^34^. There was the most variability in PSI among the 76 different tumors for IR compared with other types of AS. From highest variation to lowest, the order was IR (average s.d. = 0.13) > ES ~ ME (0.1) > A3 (0.09) > A5 (0.08). Mutually exclusive exons had only 135 variable events with s.d. > 0.06, and thus, were not considered for clustering. There was some overlap among the genes corresponding to the most variable events for the five AS types considered (Supplementary Fig. 1a).

**Fig. 1:**
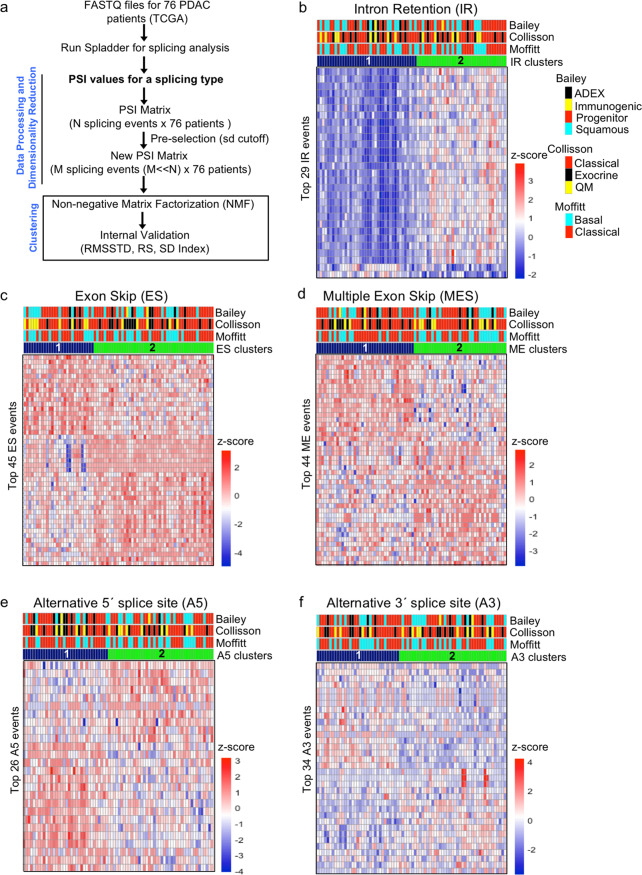
Clustering of PDAC patients based on AS events. **a** Workflow for NMF clustering of PDAC patients. PSI values for five types of AS events (intron retention, exon skip, multiple exon skip, and alternative 5′ and 3′ events) were obtained for 76 high-purity patients using Spladder^34^. The most variable events for each AS type were used for NMF clustering. The resulting clusters were compared for compactness and separation. **b**−**f** Heatmaps comparing AS levels between two patient clusters based on intron retention (**b**), exon skip (**c**), multiple exon skip (**d**), alternative 5′ splice site (**e**), and alternative 3′ splice site (**f**). Two clusters for each AS type were generated using the workflow shown in (**a**). Each column represents one patient and each row represents one of the top NMF events for that AS type. Top NMF events were obtained using the criteria described by Kim et al.^96^. PSI values were transformed into *z*-scores that are color-coded such that higher inclusion levels are shown in red and lower inclusion levels are shown in blue. Upper bars above the heat indicate the assignment of each patient to gene expression-based PDAC subtypes published by Collisson et al.^12^, Moffitt et al.^13^, and Bailey et al.^14^ The lowermost bar above the heatmaps represents assignment of patient clusters to a particular AS type (shown in blue and green).

**Fig. 2:**
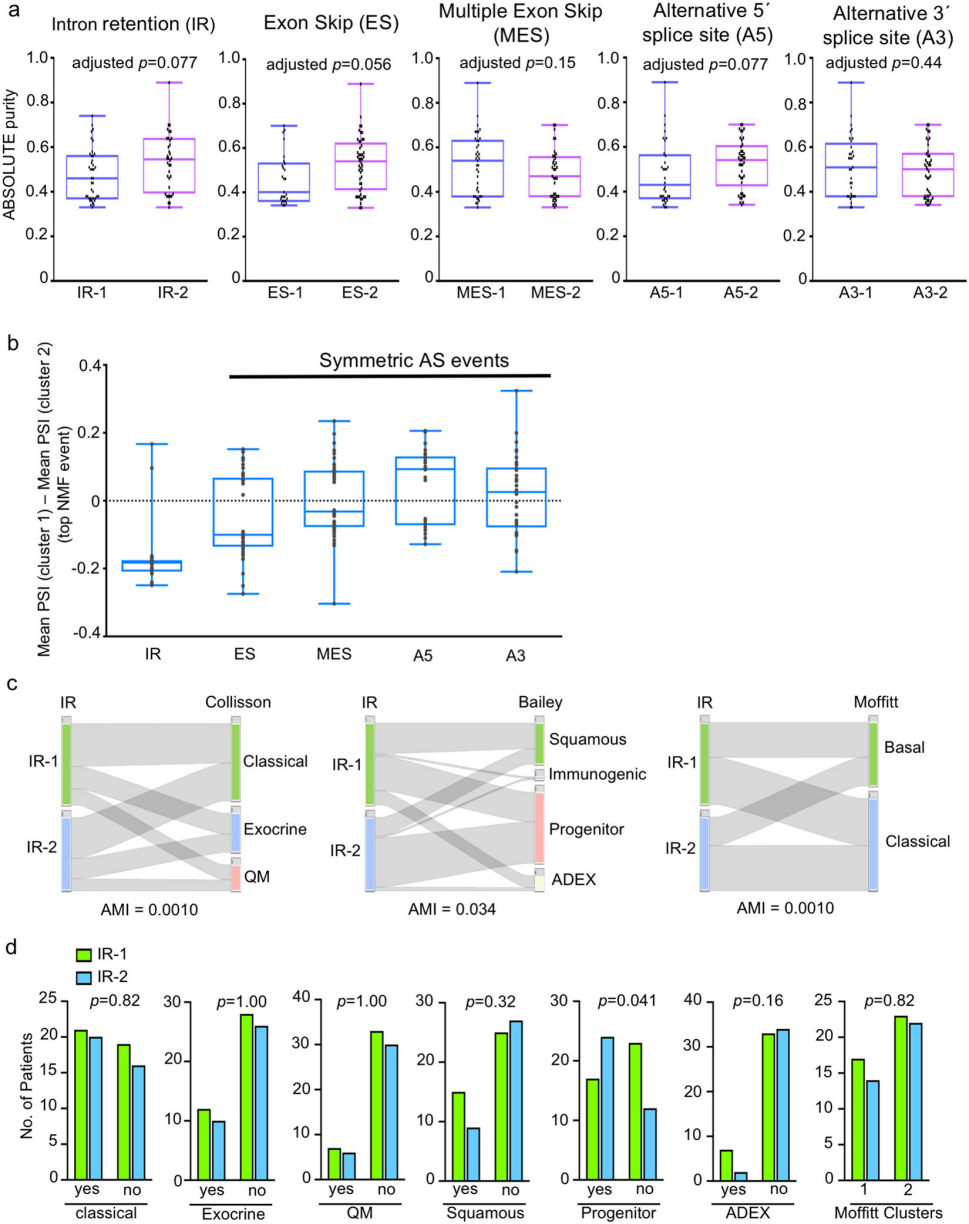
Characteristics of PDAC clusters developed based on five splicing types. **a** Box plots comparing the purity of tumors (based on the ABSOLUTE method^39^) from patients assigned to the different splicing-based clusters. Two clusters (1 and 2) for each of the five splicing types (IR, ES, MES, A5, and A3) were generated using the non-negative matrix factorization (NMF) algorithm^35^. *p* values comparing tumor purities were calculated using the two-tailed Mann−Whitney *U* test and adjusted for multiple testing correction (Benjamini−Hochberg method). Box plots in (**a**) and (**b**) show the 25th and 75th percentiles, median, and whiskers that extend to the minimum and maximum values. **b** Boxplot showing the distribution of differences between the mean PSIs for clusters 1 and 2 for all top NMF events for the indicated AS type. The number of top NMF events for each splicing type is shown in parenthesis. The position at which PSI for cluster 1 minus the PSI for cluster 2 equals zero is shown as a dashed line. The distribution of difference values is considered “symmetric” if the median of the distribution is close to zero. **c** Comparison of the classification of tumors based on intron retention (IR) clusters from this study with the classifications previously reported based on gene expression clusters by Collisson et al.^12^, Bailey et al.^14^, and Moffitt et al.^13^ Plots were generated using StratomeX visualization tool^123^. Values for adjusted mutual information (AMI) that indicate the extent of similarity of the two cluster groupings are shown. **d** Bar plots showing the classification of PDAC tumors into gene expression-based clusters for each of the IR clusters. *p* values from two-tailed Fisher’s exact tests are shown. QM quasi-mesenchymal. ADEX aberrantly differentiated endocrine exocrine.

Unsupervised clustering of 76 PDAC patients was performed using the most variable AS events identified based on the standard deviation cutoff for each splicing type: ES, 573 events (s.d. > 0.08); IR, 565 events (s.d. > 0.1); A5, 595 events (s.d. > 0.06); A3, 602 events (s.d. > 0.06); and MES, 559 events (s.d. > 0.07) (Supplementary Data 1). PDAC samples were clustered for each of the five splicing types based on PSI scores for the most variable events (Supplementary Data 1). We used the non-negative matrix factorization (NMF) method^35^ according to the workflow in Fig. 1a. NMF clustering yielded two clusters for each of the five splicing types as described previously^36^ (Fig. 1b–f, Supplementary Fig. 1b and Supplementary Data 2). The clusters from each splicing type were evaluated for cluster compactness (root mean square standard deviation (RMSSTD) and *r*-squared (RS)) and cluster separation (SD validity index)^37,38^. Among the five types of AS events, the clusters generated based on IR events (IR-1 and IR-2) exhibited the best scores for two out of three types of validation metrics (maximum value for RS and minimum value for SD validity index) (Supplementary Data 3). Consistent with the higher quality clusters generated based on IR, principal component analysis revealed that the principal component that best separates the two clusters explains the most variance for clusters generated by IR (68.5% for IR vs 30.3% or less for other types of AS) (Supplementary Fig. 1b). For RMSSTD, the A5 clusters scored the best (minimum value out of all splicing types). The tumors belonging to patients in the two clusters for all AS type were not significantly different (adjusted *p* > 0.05) in terms of the ABSOLUTE^39^ purity scores (Fig. 2a), which suggests the clusters defined by AS events are likely associated with cancer cells rather than other cell types in the sample. Our data thus identify two distinct clusters of PDAC patients based on differences in the pattern of IR in the tumor cells.

For all splicing types except IR, each of the two clusters exhibit AS changes that were “symmetric” or “balanced,” with some genes with higher PSIs and some genes with lower PSIs in the same tumor (Figs. 1 and 2b). For these splicing types, the difference in the mean PSIs between clusters 1 and 2 is approximately 0 (Fig. 2b). In contrast, for IR, the pattern of AS events was strongly “asymmetric” in that higher intron retention levels for the vast majority of genes were present in the tumors in cluster 2. The strikingly different pattern of IR in the two IR-based PDAC clusters coupled with better cluster validation metrics suggests that IR could be a metric of PDAC heterogeneity.

Comparison of AS-based clusters with clusters previously determined based on gene expression by Collisson et al.^12^, Moffitt et al.^13^, and Bailey et al.^14^ did not reveal significant concordance between the AS-based clusters and any of the previously reported classifications (Figs. 1b–f, 2c, d and Supplementary Data 4). Adjusted mutual information (AMI) scores comparing AS with gene expression clusters were 0.0010, 0.034, and 0.0010, for IR-Collisson, IR-Bailey, and IR-Moffitt comparisons, respectively (Fig. 2c and Supplementary Data 4). The only significant overlap between the IR clusters and the gene expression-based clusters (*p* > 0.05, two-tailed Fisher’s exact test) was overlap between the progenitor (Bailey classification) and IR-2 cluster (41 and 59% overlap of progenitor cluster with IR-1 and IR-2 clusters, respectively, *p* = 0.041) (Fig. 2d). These results indicate that AS (including IR) could represent an independent predictor of pancreatic cancer tumor-to-tumor heterogeneity that is distinct from the basal/squamous versus classical/pancreatic progenitor classification previously described. The IR clusters also did not show a high concordance with any of the clusters generated based on other AS types with both AMI and adjusted Rand index values ranging from 0.02 to 0.3 (Supplementary Fig. 2a and Supplementary Data 4). The best concordance was found between IR and A5 clusters (AMI = 0.03) (Supplementary Fig. 2b). To test the robustness of the IR clusters to the clustering method, we used *k*-means and hierarchical clustering methods to generate two clusters in addition to NMF. The two IR clusters determined by *k*-means or hierarchical clustering (Supplementary Data 2) agreed well with the IR-1 and IR-2 clusters obtained using the NMF clustering algorithm (NMF vs. *k*-means, AMI = 0.71 and adjusted Rand index = 0.80; NMF vs. hierarchical, AMI = 0.60 and adjusted Rand index = 0.66). We also used the Whippet algorithm^40^, as an alternative to Spladder, to determine IR events, followed by NMF clustering to form two clusters of tumors (Whippet-1 and Whippet-2). We found that Whippet-1 and Whippet-2 had high concordance with IR-1 and IR-2 (AMI and adjusted Rand index values of 0.71 and 0.75, respectively) (Supplementary Fig. 2c). Overall, our data, taken together, demonstrate that intron retention is a novel and robust dimension for generating PDAC subtypes that are distinct from those previously described.

### Clinical properties of AS clusters

**Fig. 3:**
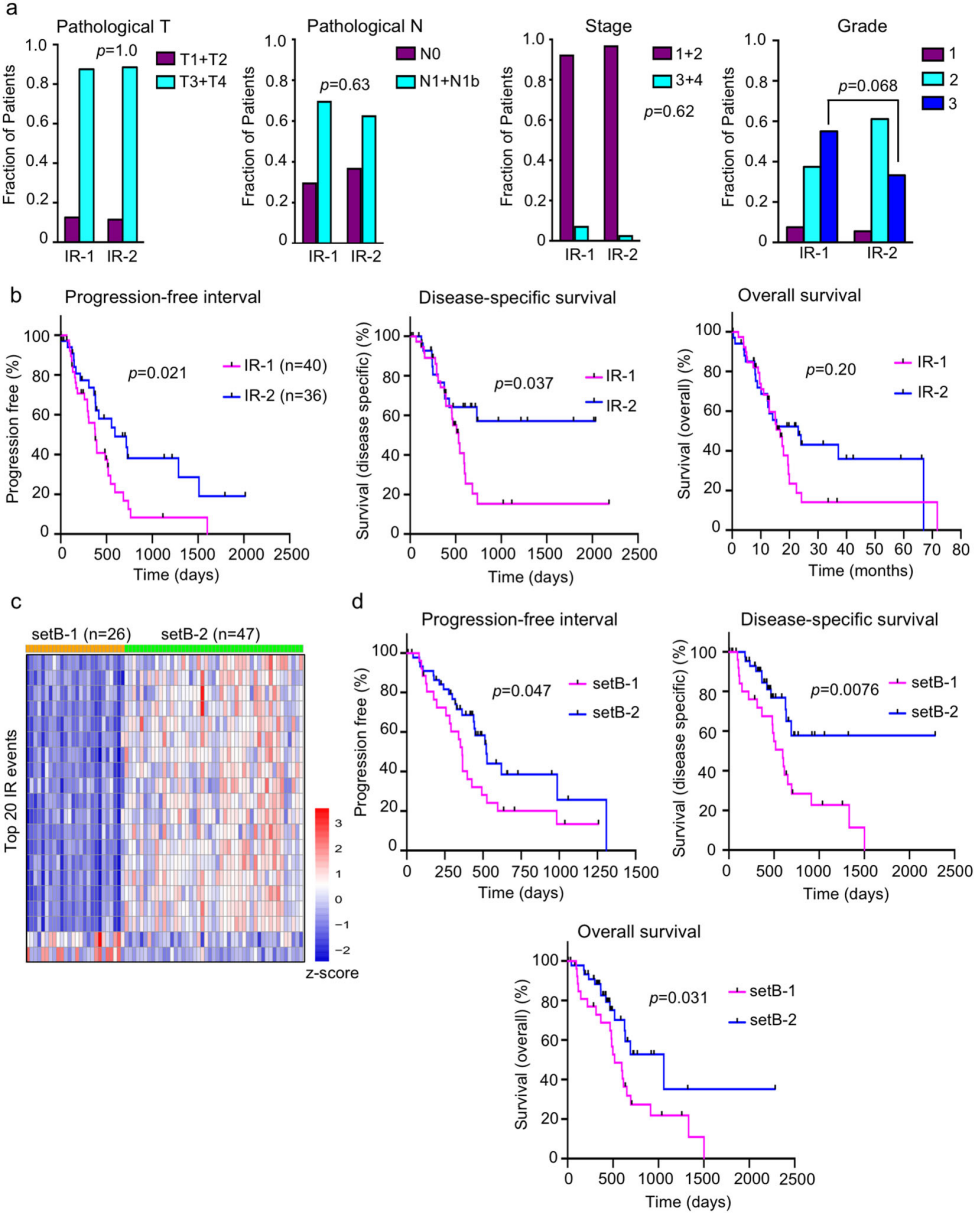
Clinical outcome and features of two intron retention (IR) clusters of PDAC. **a** Comparison between the two IR clusters and clinical measures of pathological T, pathological N, stage, and tumor grade (detailed definitions of the clinical parameters can be found in Katz et al.^124^ and https://www.cancer.org/cancer/pancreatic-cancer/detection-diagnosis-staging/staging.html). For all comparisons, the *p* value from a two-tailed Fisher’s exact test is shown. **b** Plots for progression-free interval, disease-specific survival, and overall survival for the two patient clusters (IR-1 and IR-2) based on intron retention events. **c** Heatmap showing the setB-1 and setB-2 clusters obtained by clustering a 73-patient cohort based on the features that distinguish IR-1 from IR-2. Each column represents one patient and each row represents one of the top NMF IR events. Top NMF events were obtained using the criteria described by Kim et al.^96^. PSI values were transformed into *z*-scores that are color-coded such that higher inclusion levels are shown in red and lower inclusion levels are shown in blue. **d** Clinical outcome differences between setB-1 and setB-2 clusters. The *p* value from the log-rank (Mantel−Cox) test is shown for each comparison. The number of patients is denoted by *n*.

The assignment of PDAC tumors to AS clusters was not significantly associated with age or sex (Supplementary Fig. 3a, b), or with clinical features such as stage, pathological T, pathological N, or grade (*p* ≥ 0.05, two-tailed Fisher’s exact test) (Fig. 3a and Supplementary Data 4) except that MES cluster 2 had a higher fraction of grade 3 patients than cluster 1 (*p* = 0.0002). The IR-1 clusters included a higher fraction (55%) of patients with the highest grade (grade 3, highly abnormal appearance of cancer cells) compared to IR-2 (33%), but the difference was not significant (*p* = 0.07, two-tailed Fisher’s exact test) (Fig. 3a, rightmost panel).

We next sought to understand whether the patient clusters generated based on AS were associated with differences in clinical outcome. Dividing the 76 tumors in this dataset based on any of the previously reported gene expression-based classifications did not result in significant differences with regard to progression-free interval (PFI). To extend this analysis, we also considered additional endpoints and found that gene expression-based clusters did not result in significantly different outcomes with regard to disease-specific survival (DSS) or overall survival (OS), except that the tumors in the Collisson quasi-mesenchymal category had worse outcome compared with the Collisson classical tumors for DSS (*p* = 0.0086) and OS (*p* = 0.0022), a difference that may reflect the activity of the nontumor stroma (Supplementary Data 4). In contrast, the clusters developed based on IR events did exhibit differences in PFI, which was our primary metric for measuring clinical outcome. We discovered that patients in IR-1 have significantly shorter PFI^41^ compared to patients in IR-2 (*p* = 0.021, log-rank (Mantel−Cox) test) (Fig. 3b, left panel). This difference in PFI outcome between the IR clusters was still significant even after accounting for age and sex (*p* = 0.021, multivariate Cox regression analysis). The hazard ratio was 0.49 indicating that being in IR-2 reduces the risk of poor outcome by 51%. Having established that there is a difference in PFI between these two groups of patients, we explored the clinical data further by performing some related analyses to gain a better understanding of the clinical significance of the difference in PFI. Patients in IR-1 exhibited worse DSS than patients in IR-2 (*p* = 0.037) (Fig. 3b, middle panel), while there was no difference between patients in IR-1 vs IR-2 for OS (*p* = 0.20) (Fig. 3b, right panel). For classification based on the other four AS types (ES, MES, A5, and A3), no significant differences in PFI was found between the two clusters (Supplementary Fig. 3c). The OS and DSS outcomes for the cluster comparisons were also not significant for these other four AS types (Supplementary Data 4). Our results show that the robust and asymmetric clusters generated based on intron retention also differ in clinical outcomes.

To further test the significance, robustness and reproducibility of our findings, we applied our IR-based PDAC tumor classification to a separate PDAC cohort containing a group of 73 low-purity tumors (ABSOLUTE purity < 33%) isolated from 73 patients collected as part of the TCGA project.^15^ We performed NMF clustering on this independent cohort using the highly variable IR events that were used for the determination of clusters IR-1 and IR-2. This yielded two clusters setB-1 and setB-2 that showed asymmetric IR pattern similar to the asymmetric pattern observed in IR-1 and IR-2 (Fig. 3c). To assess whether the IR-based clusters in this independent set of PDAC patients affects clinical outcome, we determined PFI for these two clusters and discovered that there was a significant difference (*p* = 0.047) between them, with patients with tumors with more efficient intron removal (setB-1) having shorter PFIs. We extended our clinical analysis and discovered that DSS (*p* = 0.0076) and OS (*p* = 0.0031) were also significantly shorter for patients in setB-1 (Fig. 3d). The results, taken together, support intron retention as a novel and independent predictor of pancreatic cancer progression.

### Differential intron retention events between IR-1 and IR-2 are overrepresented in splicing factors and oncogenes

**Fig. 4:**
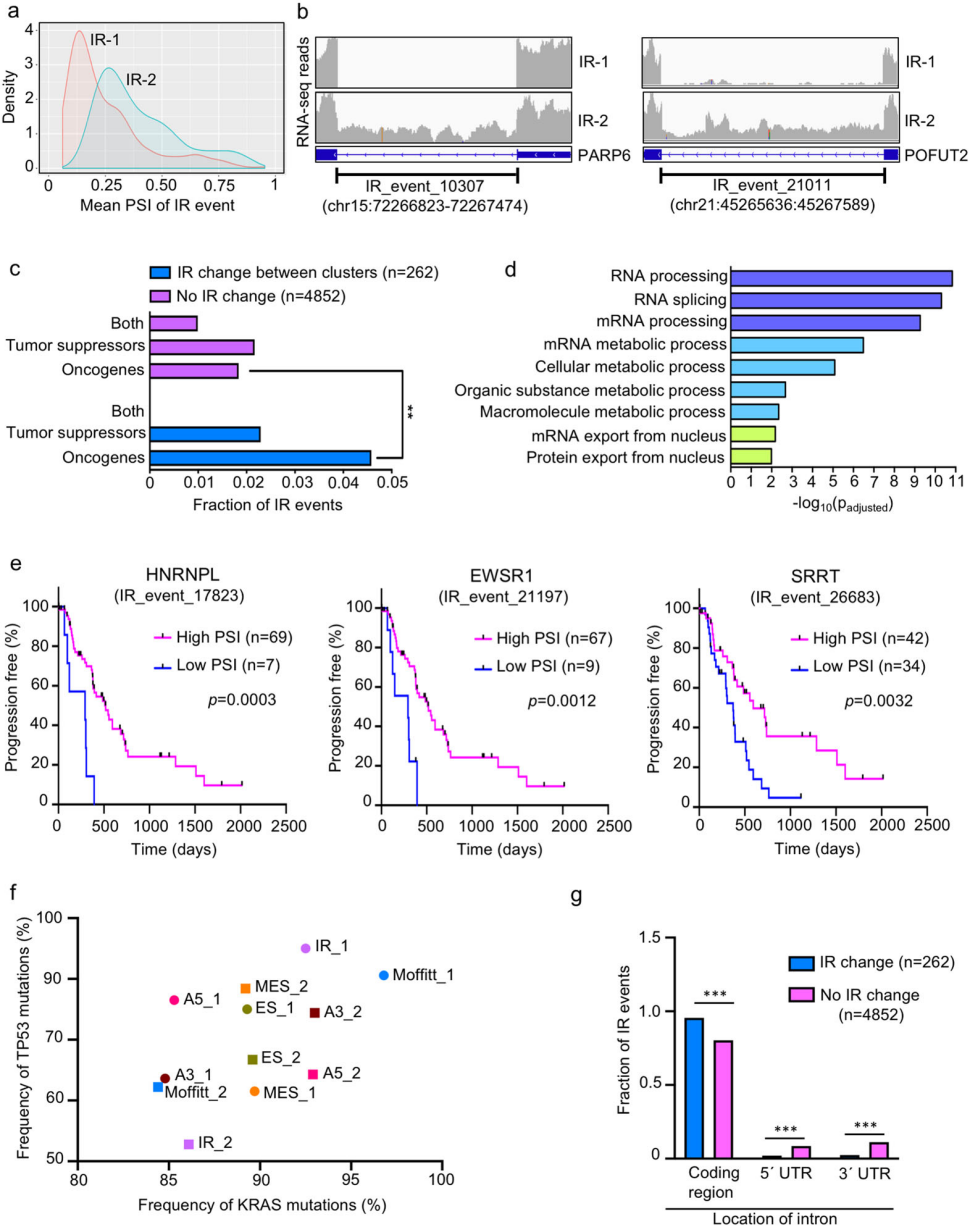
Differential IR events between the two IR-based PDAC clusters. **a** Density plots comparing mean PSI values for the 262 IR events that define IR-1 and IR-2. **b** Integrated Genomics Viewer^125^-based representation of IR events for PARP6 and POFUT2 showing higher read coverage (higher levels of retained introns) in IR cluster 2 (lower track) compared to IR cluster 1 (upper track). Coordinates (hg38 human genome assembly) of the retained intron are also shown. IR event numbers correspond to the designation in the Spladder^34^ differential splicing program output (Supplementary Data 5). **c** Comparison of IR events that changed significantly between the two IR clusters (total 262 events) with those that did not change (total 4852 events) for genes assigned as oncogenes, tumor suppressors, or both only (OncoKB^115^ and COSMIC^114^). *p* values were calculated using two-tailed Fisher’s exact test. **d** Functional enrichment analysis of the genes related to 262 differential IR events as determined using g:Profiler^112^. Adjusted *p* values (*p*_adjusted_) for GO term enrichment was calculated using g:SCS^112^. Only annotated genes were used to calculate statistical significance. Enriched GO terms for molecular function and biological process are shown and bars are color-coded based on *p* value. **e** Plots for PFl comparing high PSI with low PSI groups for three significant prognostic IR events using Survminer^111^ analysis. The three IR events shown correspond to HNRNPL (a splicing factor), EWSR1 (an oncogene), and SRRT (a splicing factor). *p* values were obtained by log-rank (Mantel−Cox) test. *n* number of patients (tumors) in each group. **f** Frequencies of KRAS and TP53 mutations for splicing and gene expression-based patient clusters. Filled circles and squares of the same color are used to indicate a cluster pair. **g** Bar graph comparing the genomic locations of introns differentially retained between the two IR clusters with introns that were not significantly retained. Significance levels: *p < 0.05; **p < 0.01; and ***p < 0.001.

To better understand the poorer clinical outcome of patients in the IR-1 cluster compared with patients in the IR-2 cluster, we considered the introns that are differently retained between the two clusters. Differential splicing analysis between IR-1 and IR-2 was performed using Spladder^34^ to obtain a list of 262 introns with significantly different inclusion levels between the two clusters (Fig. 4a and Supplementary Data 5). For 260 out of 262 IR events, tumors in the IR-2 cluster, on average, have a higher fraction of transcripts that include the intron compared to tumors in the IR-1 cluster. Average read counts in the differentially retained introns are shown for two representative genes in Fig. 4b.

We sought to understand the biological functions of the genes that encode the 262 differential IR events. The fraction of IR events in oncogenes was significantly higher for the group of genes that include the 262 differentially retained introns (4.6%) compared to a group of 4852 IR events with no change in levels between IR-1 and IR-2 (1.8%) (*p* = 0.005, two-tailed Fisher’s exact test) (Fig. 4c and Supplementary Data 5). All oncogene-related introns in the 262-intron set (corresponding to 12 oncogenes) had a higher fraction of transcripts with retained introns in IR-2 compared to IR-1. These oncogene-producing transcripts with retained introns in IR-2 are likely to interfere with the production of functional onco-proteins due to detention of these transcripts in the nucleus, thus preventing translation, or changes in the translated protein sequence and structure due to the insertion of additional amino acids contributed by the retained intron. Oncogenes with IR events that differ between IR-1 and IR-2 tumors include EWSR1, FUS, SF3B1, and STAT6. No enrichment of tumor suppressor genes was found in genes corresponding to the 262 differentially retained introns compared to a control group of genes related to 4852 IR events (0.83, two-tailed Fisher’s exact test).

Of the 223 genes representing the 262 IR events, 212 genes were protein coding, 2 genes encoded long noncoding RNAs, and the remaining 9 were pseudogenes (Supplementary Data 5). Gene ontology (GO) analysis of the 223 genes revealed overrepresentation of GO terms associated with splicing, metabolism, and nuclear export (*p* < 0.05) (Fig. 4d and Supplementary Data 6). The overrepresentation of splicing-related genes suggests there may be feedback mechanisms in which splicing factors themselves are regulated through splicing of their own introns, as has been reported for other systems^42^.

Out of the 262 IR events, 20 were found to be individually predictive for pancreatic cancer clinical outcome, when only 13 would be expected (Supplementary Data 7). For these 20 IR events, the PFI outcomes were significantly different for tumors in the low PSI group compared with tumors in the high PSI group (adjusted *p* < 0.05) (Supplementary Data 7). For all 20 of these introns, the low PSI group was associated with worse PFI outcome compared to the high PSI group. The PFI plots for three of these predictive IR events in heterogeneous nuclear ribonucleoprotein L (hnRNPL), EWS RNA-binding protein 1 (EWSR1), and serrate, RNA effector molecule (SRRT) are shown in Fig. 4e.

### Independence of intron retention events and mutations or CNVs

To better understand the basis for the difference in survival between IR-1 and IR-2, we compared the frequency of gene mutations in tumors in the two clusters (Supplementary Data 8). Among all genes in the genome, the only gene with a significant difference in mutation frequency between IR-1 and IR-2 was the tumor suppressor gene TP53 (producing p53 protein). The frequency of TP53 mutations was significantly higher (*p* = 0.003, two-tailed Fisher’s exact test) in IR-1 (85%) compared to IR-2 (52.8%), while no significant difference in mutation frequency between the IR clusters was found for KRAS, which is frequently mutated in PDAC (92.5% vs. 86.1%; *p* = 0.46). The TP53 and KRAS mutation frequencies were not found to be significantly different between the two clusters obtained for other splicing types (ES, ME, A5, and AE) or for two gene expression-based clusters^15^ that differentiate two PDAC subtypes by Moffitt et al.^13^ (Fig. 4f). Patients with tumors that contain TP53 mutations presented with tumors of higher grade (*n* = 28, grade 3) than patients with tumors with no TP53 mutations (*n* = 6, grade 3) (*p* = 0.044, two-tailed Fisher’ exact test). The PFI and DSS outcomes between the IR clusters remained significantly different when taking into account TP53 mutation status (*p* values of 0.040 and 0.046, respectively, multivariate Cox regression analysis), but the difference in outcome was less stark, suggesting it is possible that p53 mutations are among the factors that contribute to the differences in clinical endpoints between IR-1 and IR-2. Among the 223 genes corresponding to 262 IR events, only 21 genes were mutated in any of the tumors in either IR-1 or IR-2 (Supplementary Data 9). For those genes with mutations, the mutation frequencies were below 6% and there were no significant differences in the mutation frequencies for these genes between the clusters. No mutations were detected in the PDAC tumors for the remaining 202 genes (Supplementary Data 9). The results support the conclusion that genes undergoing differential intron retention between the two clusters are not significantly affected by mutations, and that mutations in splice sites are unlikely to explain the differences in intron retention. None of the copy number variants (CNVs) associated with any of the 223 genes (Supplementary Data 9) were present at significantly different frequencies between the two IR clusters (*p* ≥ 0.05, two-tailed Fisher’s exact test). Thus, IR events in PDAC do not seem to be associated with genetic changes such as mutations or CNVs, with the possible exception of TP53 mutations.

### Association between intron retention events and gene expression

Retained introns are expected to affect a transcript’s fate differently depending on their location within the transcript. IR events in coding sequences are associated with detained introns and nonsense-mediated decay as a result of premature termination codons^43^, while retained introns in 5′ and 3′ UTRs are associated with altered translation or transcript stability, respectively^44^. Comparing the 262 introns that were differentially retained between the two IR clusters with 4852 introns that were not differentially retained showed that the differentially retained introns were significantly more likely to be present in coding regions (*p* < 0.0001, two-tailed Fisher’s exact test), while introns in the control set were more likely to be found in 5′UTR and 3′ UTR regions (Fig. 4g).

The enrichment for IR events in coding regions distinguishing PDAC clusters IR-1 and IR-2 suggested that the intron retention events may affect expression levels of the associated genes. To test whether the genes undergoing differential retention of introns also change in gene expression between the two clusters, we performed differential gene expression analysis comparing the two IR clusters (Supplementary Data 10). There were 7152 genes differentially expressed between IR-1 and IR-2 (adjusted *p* < 0.05), with only 344 genes upregulated and 1707 genes downregulated in IR-1 by twofold or more (Supplementary Data 10). Gene ontology (GO) analysis of the genes differentially expressed between IR-1 and IR-2 showed that upregulated genes in IR-2 were enriched in splicing factors, raising the possibility that, for these splicing factors, the increase in gene expression in IR-2 is contributed by transcripts with retained introns, as intron retention events were overrepresented in splicing factors in IR-2 (Supplementary Fig. 4a and Fig. 4d). Out of the 223 genes undergoing differential retention of introns, 134 genes involving 164 IR events showed significant expression changes between the clusters (0.9 ≤ absolute fold change ≤ 2.1) (Supplementary Data 10 and Supplementary Fig. 4b). Among these 134 genes, 132 were downregulated and also showed lower intron retention levels in IR-1. Out of these 132 genes, five were oncogenes (TYK2, SRSF2, IKBKB, STAT6, and MST1R). The changes in gene expression of these 134 genes were significantly correlated with the change in the percent spliced in (ΔPSI_mean_) for the IR event in the same gene (Spearman correlation coefficient = 0.4, *p* < 0.0001) (Supplementary Fig. 4c). The fact that expression levels are lower for genes in the IR-1 tumors in which introns are consistently removed suggests the mechanism is unlikely to be nonsense-mediated decay, which would result in higher expression when the introns are removed. Further, none of the core nonsense-mediated decay genes are significantly differentially expressed in IR-1 vs. IR-2 (Supplementary Data 10). These results would be consistent with a model in which the higher levels of intron removal in genes in IR-1 results in more efficient export of transcripts from the nucleus, while the reduced levels of intron removal in IR-2 results in transcripts that are detained in the nucleus and accumulate. These accumulated transcripts in IR-2 could contribute to higher expression for these genes.

### Role of RBPs in differential intron retention between IR clusters

**Fig. 5:**
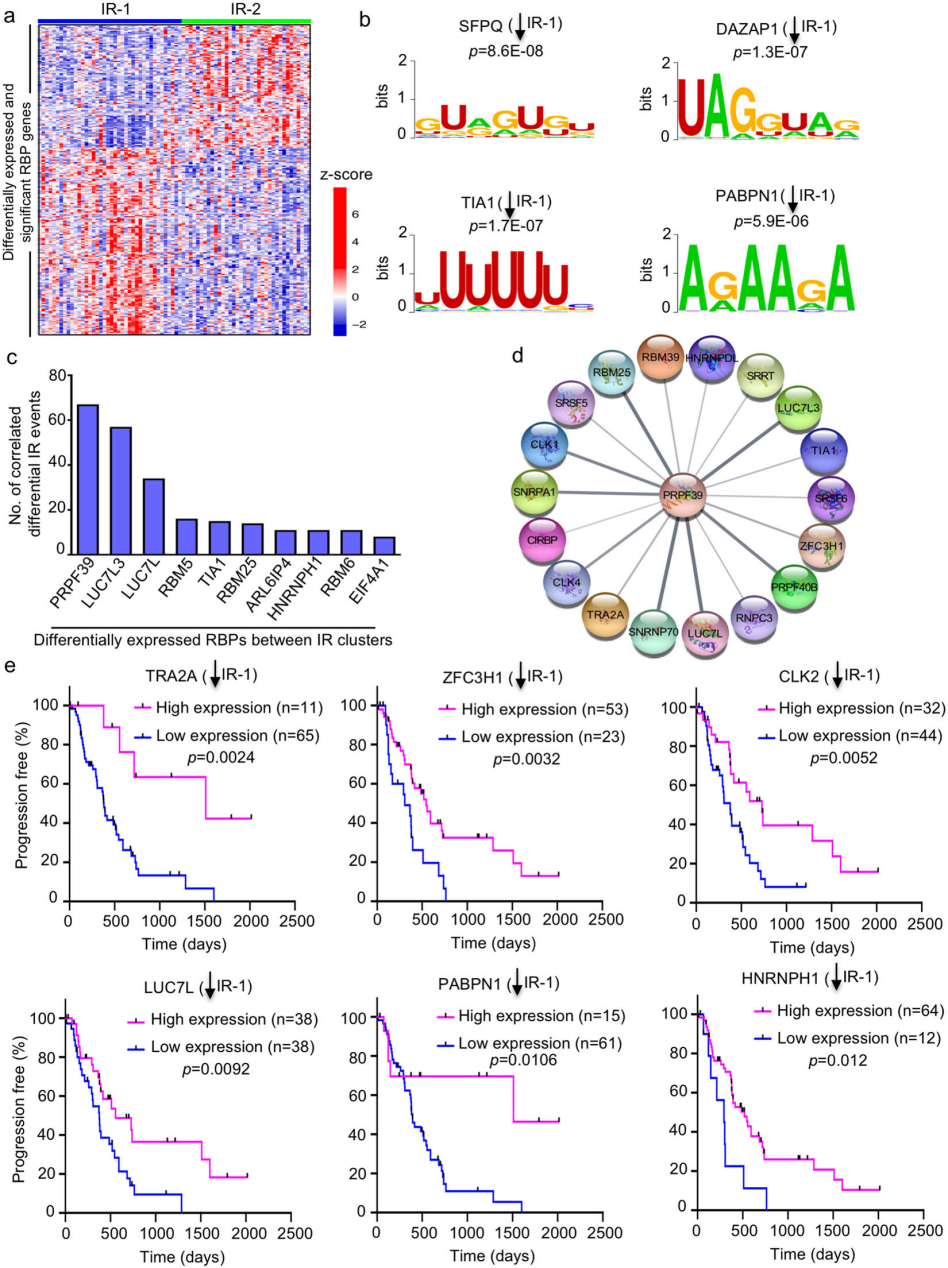
RBP genes that differentiate the two IR clusters. **a** Gene expression heatmap for 258 differentially expressed RBP genes between the two IR clusters. *Z*-score values (calculated from normalized log_2_[counts]) shown in red and blue indicate high and low expression, respectively. **b** Sequence logos of the RNA motifs that are significantly enriched in the 262 differentially retained introns between the two IR clusters. The RBPs corresponding to these motifs also significantly change in expression between the two IR clusters. The *y*-axis represents bits of information^126^. *p* values (one-tailed Fisher’s exact test) from the AME motif enrichment analysis^116^ (MEME suite^117^) are also shown. **c** Top differentially expressed RBPs between IR-1 and IR-2 whose expression levels are highly correlated (correlation coefficient ≥ 0.7) with the PSI values for the maximum number of IR events. **d** Interacting proteins of PRPF39 as determined by network analysis of RBPs that are upregulated with IR-2. Network analysis was performed on Cytoscape^108^ using the STRINGapp^107^. Interacting proteins are shown as outer circles connected by a line to the central circle (PRPF39). **e** Plots comparing PFI of the high- and low-expression groups for the prognostic RBPs obtained from the Survminer^111^ analysis. *p* values from log-rank (Mantel−Cox) test are shown; *n* number of patients (tumors). Downward arrows indicate reduced expression of the indicated RBP in IR-1.

Differential intron retention between the IR clusters could result from differences in the expression levels of specific RBPs (including splicing factors) that bind to pre-mRNA. By comparing a list of 1565 genes that encode RBPs^45,46^ with the list of 7152 genes differentially expressed in IR-1 vs. IR-2 (Supplementary Data 10), we found 258 RBP genes expressed at significantly different levels in the two IR clusters (adjusted *p* < 0.05) (Fig. 5a and Supplementary Data 10). Out of the 258 differentially expressed RBP genes, 102 genes were downregulated and 157 genes were upregulated in cluster 1. For most of these differentially expressed RBPs (233 out of 256), there was no difference in intron retention levels between IR-1 and IR-2. In contrast, for the remaining 25 of the 258 RBP genes that were also included on the list of 223 genes undergoing differential IR between the two clusters (*p* = 2.2e−29 for the overlap, hypergeometric test), 24 out of 25 genes were downregulated in cluster 1. For these 24 RBP genes, intron retention levels and gene expression levels were both higher in IR-2 compared to IR-1^47–49^. This may reflect an increased contribution to gene expression of poorly spliced transcripts that accumulate in the nucleus in IR-2, as has been observed in other systems^47–50^. This buildup of IR-containing transcripts in the nucleus in IR-2 could lower the production of functional RBP proteins in the cytoplasm. Mutations in RBP genes can also lead to altered functions of RBPs in cancer^45^, but RBP mutations are unlikely to contribute to the differences in outcome for patients in IR-1 vs. IR-2 because mutation frequencies were not significantly different between the two IR clusters for any of the RBP genes (*p* > 0.05, two-tailed Fisher’s exact test) (Supplementary Data 8). Instead, our findings suggest a process in which IR of transcripts encoding RBPs affects their activity, and thus impacts the splicing of other genes^42^.

Next, we wanted to understand the mechanism of higher intron retention levels in IR-2 compared to IR-1. The retention or exclusion of introns during splicing may depend upon the binding of RBPs to specific RNA motifs in introns^51–53^. Motif analysis revealed 43 DNA motifs enriched in the DNA sequences surrounding the 262 differentially retained introns compared to 4852 introns that were not significantly differently retained between the two clusters (adjusted *p* < 0.05, one-tailed Fisher’s exact test) (Supplementary Data 11). These 43 motifs were related to 35 RBPs. Seven (SFPQ, DAZAP1, TIA1, PABPN1, CELF6, RBMS3, and PABPC5) of these 35 RBPs significantly change in expression between the IR clusters (Supplementary Data 11); RBMS3 and PABPC5 are upregulated, while the other five are downregulated in IR-1 (Fig. 5b). Out of these seven RBPs that change in expression between IR-1 and IR-2 and recognize motifs that are enriched in the introns, TIA1 also showed differential intron retention between IR-1 and IR-2, with greater levels of retained intron-containing TIA1 transcripts in IR-2. Genes with TIA motifs include RNA-binding proteins and oncogenes.

To gain further understanding of the potential regulation of 262 IR events by RBPs, we correlated the expression of 258 RBPs that change in expression in IR-1 vs. IR-2, with the IR levels (PSI values) of 262 introns. This led to 267 highly correlated and significant (Pearson correlation coefficient ≥ 0.7, adjusted *p* < 0.05) RBP-IR event pairs (Supplementary Data 12). These pairs were represented by 25 RBPs (Supplementary Data 12) and 139 IR events. The top ten RBPs with the highest number of highly correlated IR events (Fig. 5c) were PRPF39 (67 events), LUC7L3 (57), LUC7L (34), RBM5 (16), TIA1 (15), RBM25 (14), ARL6IP4 (11), HNRNPH1 (11), RBM6 (11), and EIF4A1 (8). All ten RBPs were significantly upregulated in IR-2 (Supplementary Data 10) and out of these, seven (PRPF39, LUC7L3, LUC7L, RBM5, RBM6, HNRNPH1, and TIA1) also had higher levels of retained introns in their transcripts in IR-2. Network analysis of the protein−protein interactions (PPI) among the RBPs upregulated in IR-2 with the STRING database^54^ showed that PRPF39 interacts with LUC7L3, LUC7L, TIA1, and RBM25 (Fig. 5d). Among these interactors, the motif for TIA1 was found to be enriched in differentially retained introns between the clusters (Fig. 5b). Binding of TIA1 to its motif could serve as a platform for recruiting other RBPs through PPIs. The TIA-LUC7L3-LUC7L-PRPF39-RBM25 protein complex formed would then affect intron retention as this complex associates with U1 snRNP to mediate splicing at 5′ splice sites^55–59^. Consistent with this model, knockdown of TIA1 results in more retained introns^60^. The higher expression levels of the RBPs that form this complex (RPF39, LUC7L3, LUC7L, TIA1, and RBM25) in IR-2 could result from increased intron retention levels of their transcripts and detention of these IR-containing transcripts in the nucleus in IR-2. This would lead to lower levels of functional proteins for these RBPs in IR-2, which could potentially disrupt the PPI among these factors. These findings, in conjunction with previous studies showing that the members of this complex can facilitate intron removal by interacting with U1 snRNP^55,56^ for 5′ splice-site recognition, suggest a possible role for PPIs among RBPs in regulating the intron retention events in IR-2.

To further understand the regulation of these highly correlated RPBs, we identified the transcription factors (TFs) that regulate this set of 258 RBPs using the ChEA3 program^61^ (Supplementary Data 13). POU5F1 was found to be the most significantly upregulated (~3-fold) TF gene in IR-2 and was associated with 10 (EIF4A1, HNRNPDL, TRA2A, RBM5, SRSF11, TIA1, ZFC3H1, PTBP2, POU5F1, and PRPF39) out of 25 RBPs that were highly correlated with IR events. The transcription factor p53 was significantly more likely to contain a mutation in tumors in IR-1 than tumors in IR-2. Among the 258 RBPs, 62 (24%) were known transcriptional targets of p53 ^62^ (Supplementary Data 13). Out of these 62 RBPs, 46 were upregulated in IR-1 and 16 were upregulated in IR-2. The upregulation of more p53 targets in tumors in which p53 is mutated and expected to be inactivated makes it less likely that p53 transcriptional induction is a key factor in regulating RBP levels.

For 63 out of 258 RBP genes, classifying the tumors into low- and high-expression groups with the Survminer package resulted in groups with significantly different PFIs (Supplementary Data 14). Except for three RBPs (SNRNP48, DDX6, and ZC3HAV1) out of the 63, the high-expression tumor group had worse clinical outcome for RBPs that were upregulated in IR-1. Likewise, the outcome was worse for the low-expression tumor group for the RBPs that were downregulated in IR-1. The PFI outcomes for six representative predictive RBPs are shown in Fig. 5e. Five (TRA2A, ZFC3H1, CLK2, LUC7L, and HNRNPH1) out of six of these RBPs were also on the list of 25 highly correlated RBPs. All six RBPs were significantly downregulated in IR-1 with a worse outcome associated with the low-expression group.

### Intron retention-based clustering of patients from other cancer types

**Fig. 6:**
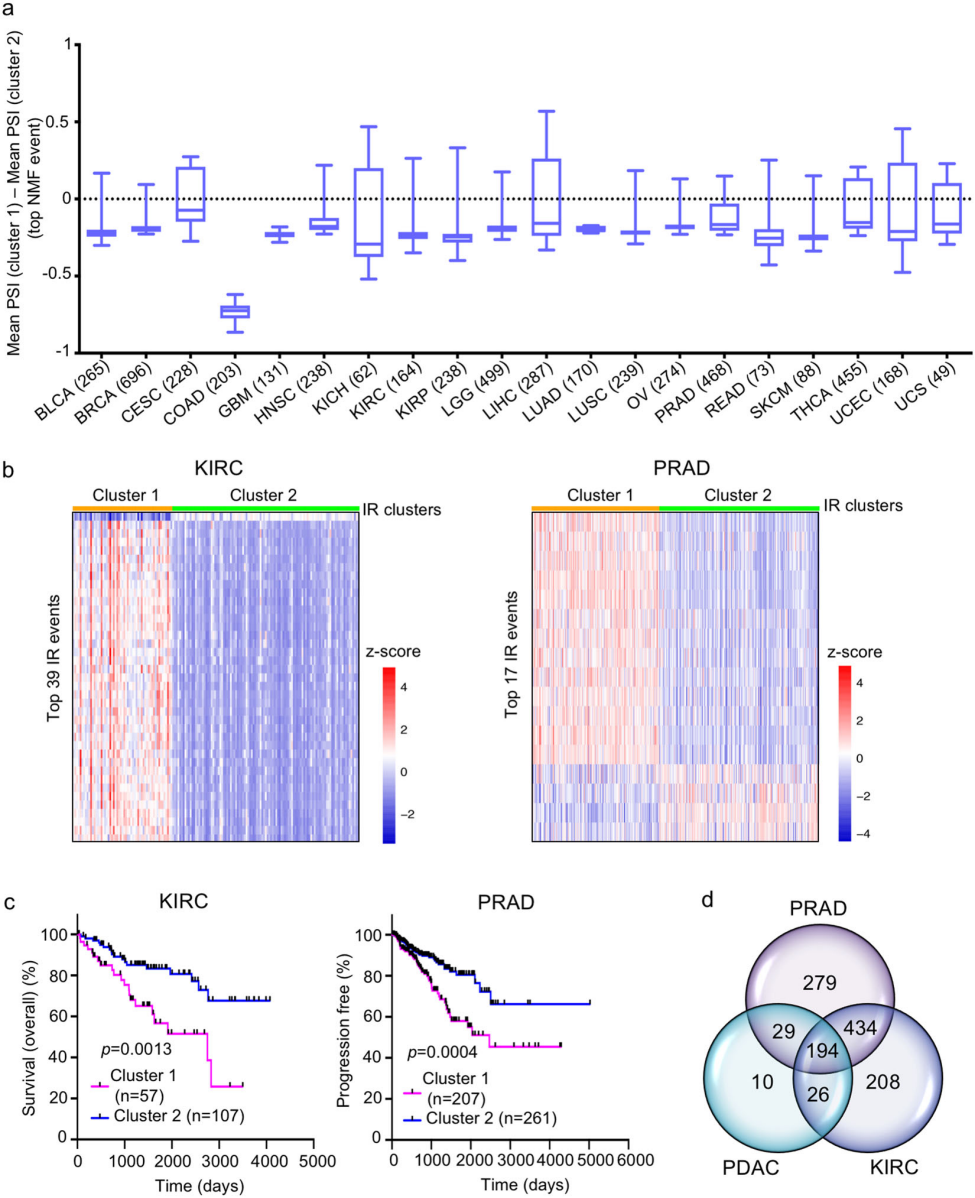
Clustering of patients of different cancer types based on IR events. **a** Box plots for the mean PSI value difference for top NMF events between the two IR-based clusters of different cancer types. The number in parenthesis indicates the number of patients for that cancer type. **b** Heatmaps comparing the IR levels of top NMF events between the two clusters of KIRC and PRAD cancer types. *z*-scores (calculated using the PSI values) are represented by red and blue colors indicating high and low IR levels, respectively. **c** OS and PFI plots comparing the two clusters of KIRC and PRAD, respectively. *p* values were determined by log-rank (Mantel−Cox) test; *n* number of patients (tumors). **d** Venn diagram showing the overlap between the three groups of RBP genes that significantly change in expression between the two clusters of PDAC, PRAD, and KIRC, respectively.

To test whether IR events can be used to generate clinically relevant clusters for tumors other than PDAC, we analyzed data for 20 other tumor types available through TCGA. For each tumor type, we identified the most variable IR events (s.d. > 0.1) and used these to cluster the patients. For most of the tumor types (15 out of 20), we obtained two clusters that were “asymmetric” (patient tumors in cluster 1 had greater mean PSI for >70% of the top NMF events compared to cluster 2 or vice versa) (Fig. 6a and Supplementary Data 15). These results demonstrate that an asymmetric distribution of IR events is a general feature of inter-tumor heterogeneity. For kidney renal clear cell carcinoma (KIRC), the two patient clusters generated by intron retention had a statistically significant difference in OS (*p* = 0.0013, log-rank (Mantel−Cox) test), while for prostate adenocarcinoma (PRAD) (Fig. 6b, c), patients in the two clusters generated by intron retention differed significantly for PFI (*p* = 0.0004) (Fig. 6b, c).

Differential gene expression analysis between the IR clusters for KIRC and PRAD revealed 862 and 936 RBP genes, respectively, with significantly different expression between the clusters (Supplementary Fig. 4d and Supplementary Data 16 and 17). Comparison of the sets of RBP genes differentially expressed in the intron retention-based clusters from PDAC, PFAD and KIRC identified 194 RBP genes that were common among the three sets (Fig. 6d and Supplementary Data 17). This common set of RBPs includes 23 out of 25 highly correlated RBPs for PDAC, suggesting involvement of similar RBP networks in all three cancer types. In all three tumor types, the tumor cluster with increased intron removal was also the cluster with lower expression levels of these RBPs. These results suggest that IR in general, and a network of specific RBPs that coordinate these IR events, are important contributors to tumor-to-tumor variation for multiple cancer types.

## Discussion

Cancer has traditionally been viewed as a result of an accumulation of mutations. During years of tumor growth and development, subsets of cells with inherited, genetic alterations clonally expand and pass these mutations to progeny cells. With time, mutations and other genetic alterations accumulate and confer upon the tumor additional properties, such as an ability to degrade the basement membrane, recruit vasculature, alter metabolic programs, and avoid senescence and apoptosis that contribute to a tumor’s aggressivenes^23^. However, accumulating data suggests that this model fails to fully capture the complexity of tumorigenesis^63^. Splicing events may represent an alternative route to tumorigenesis. In multiple tumor types, splicing has been reported to outperform gene expression analysis in predicting survival^64^. Specific AS events are prognostic for different cancer types such as cervical cancer^65^, endometrial cancer^66^, soft tissue sarcoma^67^, hepatocellular carcinoma^68^, and colorectal cancer^69^. Mutations in splicing factors are being recognized as important oncogenic events^26–28^. Exome sequencing of chronic lymphocytic leukemia, for instance, revealed that 10–15% of patients have mutations in SF3B1^70,71^, which was also identified as an oncogenic driver of breast cancer^72^. Single nucleotide polymorphisms in splicing factors have been associated with the risk of pancreatic cancer^73^. Mutations at splicing sites that prevent proper splicing can also contribute to tumorigenesis^27,29^. Finally, even with full information about the mutational, copy number variation, and other genetic alterations in a tumor, there remains considerable variability in patient outcome, demonstrating that this information fails to capture all of the contributors to tumor progression.

In our study of PDAC, we found that classifying tumors based on IR events resulted in two distinct clusters with significantly different clinical outcomes. These IR-based clusters did not show any significant difference in mutational levels in the genes undergoing IR or in splicing factors. IR events are highly prevalent in the human genome; approximately 80% of protein-coding genes in humans are associated with IR events^60^. Further, IR is conserved across phyla, as IR events are common in fungi^43^, insects^43^, viruses, and plants^43,74^. Splicing events like IR that are separate from mutational events can contribute to the progression of tumors that contain few mutations^30^. Dvinge and Bradley^25^ analyzed alternative splicing events in 16 different cancer types and found asymmetric changes in IR with retention of alternative introns strongly enriched in all cancer types compared to adjacent normal tissues with the exception of breast cancer where the adjacent tissue was enriched for retained introns. Other types of AS (use of cassette exons and competing 5′ and 3′ splice sites) displayed no preferential direction of change between cancer and normal tissues^25^. Dvinge and Bradley^25^ included some tumors of the gastrointestinal tract such as stomach, colon, liver, and rectal cancers, but not pancreatic cancer. Investigating prostate cancer, Zhang et al.^47^ found that splicing dysregulation driven by CNVs correlated with disease progression. Intron retention, in particular, was found to correlate with prostate cancer stemness and aggressiveness^47^. In their study, Zhang et al.^47^ also observed that intron-containing transcripts were present at higher levels in tumors than their spliced transcript counterparts. An asymmetric pattern of IR change has also been associated with changes in cellular state. For example, differentiated and quiescent cells have higher IR levels compared to proliferating cells^48,75–77^. In yeast, intron retention in the *Gcr* transcription factor has been associated with changes in nutrient status and the isoforms with and without the intron were both required for expression of target genes involved in glycolysis^78^. In agreement with previous studies in cancer tissue, we also observed an asymmetric pattern of IR between clusters of PDAC tumor samples, but not other AS types. Also, in comparing our findings in PDAC with other tumors, we found that for most tumor types (Fig. 6a), changes in IR events between tumor clusters were asymmetric. In contrast to the previous studies mentioned above, where tumors were compared with normal tissues, we compared AS events among tumors to determine whether AS events would predict patient-specific outcome. Somewhat surprisingly, while Dvinge and Bradley found that retaining introns was, in general, associated with cancer compared with normal tissue, in our studies, the patients with PDAC tumors with higher levels of intron removal (IR-1 cluster) were most likely to have aggressive disease.

**Fig. 7:**
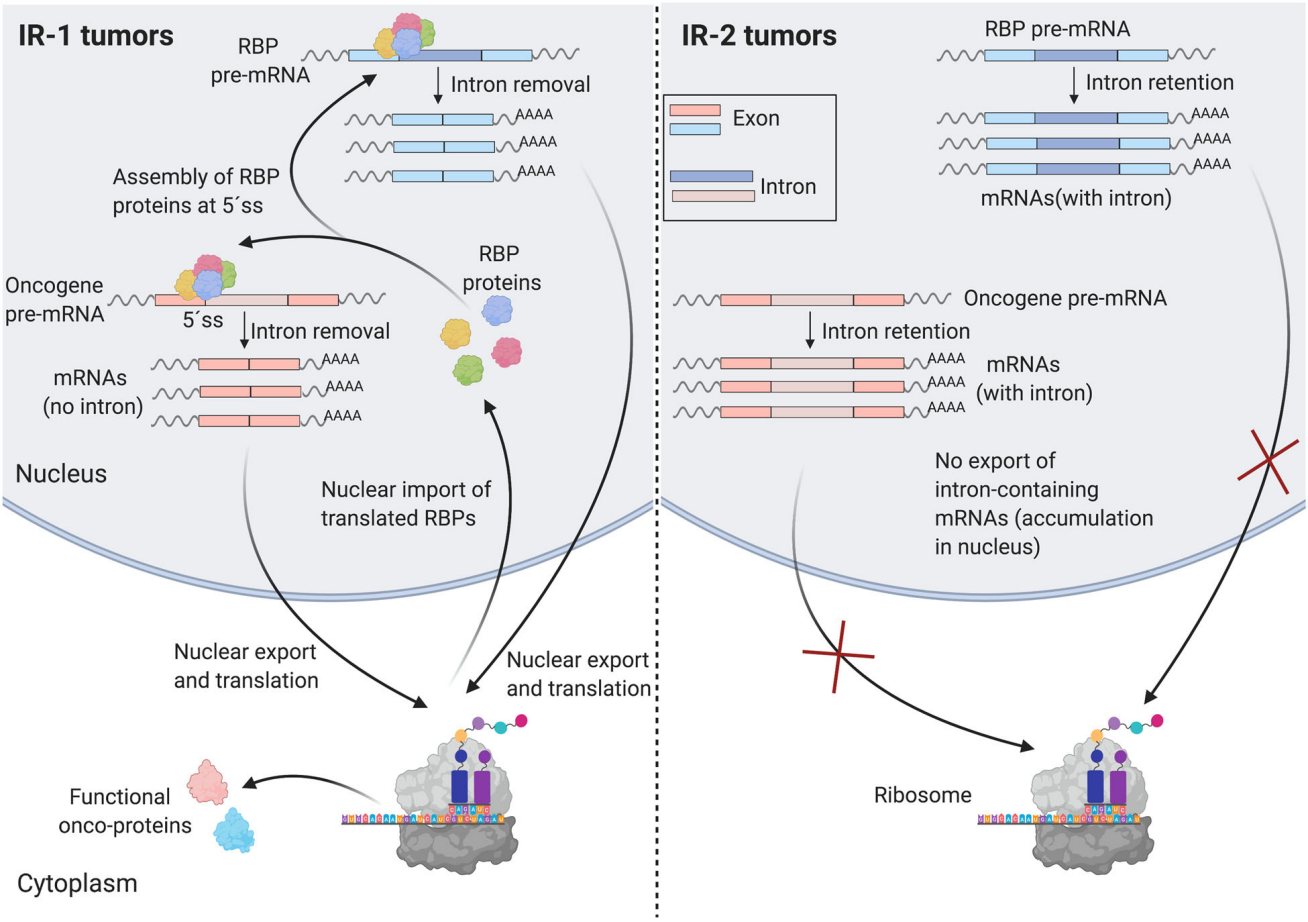
Simplified hypothesized model of the differential regulation of IR events in IR-1 and IR-2 clusters. The RBP proteins (such as PRPF39, LUC7L3, LUC7L, TIA1, and RBM25) form a complex that interacts with U1snRNP (not shown) at weak 5′ splice site (5′ ss) to facilitate intron removal. In IR-1, this RBP complex binds the 5′ ss of its own introns in a feedforward loop leading to intron removal and export of mature intron-less mRNAs to the cytoplasm where they are translated to RBP proteins. The RBP complex also removes introns from transcripts that encode oncogenes that are translated to functional onco-proteins. In IR-2, introns in the transcripts of these RBPs are retained in the mature mRNA, leading to accumulation of these intron-containing transcripts in the nucleus, causing reduced translation of these transcripts and thus a reduced level of RBP complexes in the nucleus. Lower levels of functional RBP complexes would reduce translation of these transcripts to functional onco-proteins. Created with Biorender.

In PDAC, we observed that oncogenes are enriched in the set of genes that undergo differential IR between clusters IR-1 and IR-2. These findings would be consistent with a model in which failure to remove introns in oncogenes in IR-2 results in intron-containing transcripts that are detained in the nucleus and accumulate, a finding consistent with previous studies^47–50^. This would result in lower functional levels of the encoded tumor-promoting onco-proteins in IR-2 (Fig. 7). The relatively greater levels of intron removal in IR-1, possibly resulting in higher levels of functional oncogenic proteins, could contribute to the poor survival of patients in IR-1. Oncogenes undergoing lower levels of intron retention in IR-1 compared to IR-2 include EWSR1, FUS, SF3B1, MST1R and STAT6. EWSR1 is an oncogene and splicing-based fusions involving EWSR1 rearrangements are causative for a subtype of sarcoma^79,80^. EWSR1 is involved in tumorigenesis-promoting fusions with other transcription factors^81^. Such fusion events have been observed in pancreatic neuroendocrine cancer^82^. Introns that are differentially retained in the FUS oncogene have been observed as recurrent events in cancers from many different tissues^25^, and FUS has been shown to affect proliferation of PDAC cells^83^. In particular, FUS mutations and deletions result in changes in gene expression and splicing changes, especially intron retention, affecting genes enriched in RNA-binding proteins^84^. SF3B1, a splicing factor and oncogene^31,70,71^, is consistently mutated in pancreatic cancer^85^. MST1R kinase has been shown to accelerate pancreatic cancer^86^. We did not observe significant differences in the frequency of mutations in FUS, SF3B1 or MST1R kinase between IR-1 and IR-2.

Our findings shed light on possible mechanisms that give rise to global changes in intron retention levels between the two clusters. Mutations and copy number aberrations are unlikely to contribute as there aren’t consistent changes between PDAC IR-1 and IR-2 with regard to mutations, with the exception of TP53. p53 can regulate the transcription of roughly one-fourth (24 out of 258) of the RBPs that are expressed at different levels between IR-1 and IR-2. Out of these p53-regulated RBPs, only three (LUC7L, TRA2A, and HNRNPDL) were part of the 25 highly correlated RBPs and the motif of only one RBP (RBMS3) was enriched in the set of differentially retained introns. Therefore, these p53-regulated RBPs are unlikely to play a substantial role in the control of IR events in the IR clusters (Supplementary Data 17).

Our results support an important role for a group of RBPs in regulating the levels of intron retention events between IR-1 and IR-2 (Fig. 7). This group of RBPs are expressed at higher levels in IR-2 and the transcripts produced from these RBP genes themselves undergo intron retention. These transcripts with retained introns are likely detained and accumulate in the nucleus in tumors in IR-2, leading to reduced levels of functional proteins. The RBPs in this group include three SR proteins (SRSF1, SRSF2, and SRSF5) associated with splicing. They also include RBPs that are part of a complex established to regulate splicing through interaction with U1snRNP (PRPF39, LUC7L3, LUC7L, TIA1, and RBM25)^56–59,87^, and are known to promote splicing of introns with weak 5′ splice sites. Retained introns have been shown to have weak splice sites^87^ and therefore, this RBP complex would be needed to remove introns not only from the transcripts produced from the RBP genes constituting the complex in a feedback loop, but also from the IR-containing transcripts generated from non-RBP genes, including oncogenes. This process of intron removal likely works efficiently in IR-1 to allow formation of functional RBPs and onco-proteins, resulting in worse clinical outcomes in IR-1 patients. In IR-2, disruption of this process may be responsible for lower levels of functional RBPs and onco-proteins due to inefficient intron removal. Thus, intron retention in IR-2 could act as a protective mechanism against cancer progression in PDAC. This mechanism is supported by the observation that knockdown of TIA1, a member of the RBP complex whose recognition motif is enriched in the set of 262 retained introns (Fig. 5b), results in increased intron retention^60^. Our findings are consistent with earlier data demonstrating that RBPs can interact with each other to form networks^88^, and that networks of RBPs, and co-regulation of enhancer vs. suppressor splicing factors^89^, may be important regulators of cancer progression^53,90–93^.

Although the above model supports our observations regarding differential levels of retained introns and different clinical outcomes between IR clusters, our study has important limitations. RNA-seq data from TCGA were not obtained separately for nuclear and cytoplasmic compartments, so we cannot directly test our hypothesis that the accumulation of transcripts in conditions in which they retain introns reflects their accumulation in the nucleus. There are alternative possibilities, for instance, intron-containing mRNAs may be translated in the cytoplasm in IR-2, and this could lead to the generation of neoepitopes that are exposed on the tumor cell surface^22^.

Taken together, our data support a model for tumor aggressiveness that relies minimally on DNA-based mutations or copy number variations. More consistent with our findings is a model in which changes in the levels of RNA-binding proteins and networks of RNA-binding proteins regulate their own splicing and the extent of intron retention of other genes through a feedforward loop. The RBP network we identified may be affecting the likelihood that introns are retained in hundreds of genes genome-wide, and by altering intron retention in tumor suppressors and oncogenes may determine a tumor’s course, even for tumors with the same genetic makeup. Aberrantly spliced isoforms that include retained introns, or splicing itself, may have potential as anticancer drug targets^27,94^.

## Methods

### Selection and acquisition of patient data

Access to the data used for analysis was approved by dbGAP and all relevant NIH Institutes and Centers through project ID 5849. The Cancer Genome Atlas (TCGA) obtains informed consent from patients included in their cohorts. We have adhered with all applicable federal regulations regarding human subjects protection. All RNA sequencing (bam and count files) and metadata files associated with PDAC patients used in this study (listed in Supplementary Data S2 of ref. ^15^) were obtained from The Cancer Genome Atlas (TCGA) hosted by the Genomic Data Commons (GDC) (https://portal.gdc.cancer.gov/) using the GDC Data Transfer Tool (https://github.com/NCI-GDC/gdc-client). These RNA-seq alignment (BAM) and count files were generated using GENCODE v22 gene annotation (https://gdc.cancer.gov/about-data/data-harmonization-and-generation/gdc-reference-files). Downstream clustering and analysis was conducted on high-purity tumors from 76 PDAC patients as described^15^. Purity estimates of the tumor samples were determined previously using whole-exome sequencing^15^ by the ABSOLUTE method^39^. For the 76 high-purity tumors, the ABSOLUTE purity was ≥33%. For tumors in set B, ABSOLUTE purity was <33%.

### Quantification of alternative splicing events

For sequencing data analysis, we used the GRCh38 (hg38) human genome assembly and GENCODE v22 comprehensive gene annotation (CHR) (genome and gene annotation files obtained from: https://gdc.cancer.gov/about-data/gdc-data-processing/gdc-reference-files), unless specified otherwise. Quantification of five alternative splicing types (exon skip, intron retention, 5′ alternative splice site, 3′ alternative splice site, and multiple exon skip) was conducted for each of the 76 high-purity PDAC patients via the build mode (https://spladder.readthedocs.io/en/latest/) of the graph-based Spladder toolbox (release 1.2.1)^34^. The build command (–build) was run using default parameters (see https://github.com/danjst/PDAC_2020). A confidence level of 3 (highest) was used. PSI values (the fraction of transcripts of a gene that include a specific exon or splice site^95^) were extracted for each splice type to generate patient-PSI matrices. Spladder assigns a missing value in the patient-PSI matrix as “NA” if an accurate PSI could not be computed because there were fewer than ten total spliced reads detected for that event. AS events containing missing values for any patient were filtered out prior to selecting the most variable events for NMF clustering (see next section). Quantification and verification of intron retention events was also conducted using Whippet, a graph-based AS quantification method^40^. The Whippet algorithm (https://github.com/timbitz/Whippet.jl) was run with default parameters. Events containing missing values, i.e., events assigned “NA” because there were fewer than one read, for any patient were filtered out prior to selecting the most variable events for NMF clustering.

### NMF clustering based on AS events

Unsupervised clustering of 76 high-purity PDAC patients was conducted based on the most variable splicing events (events with the most variation of PSI values across the patients) that were selected by applying a standard deviation cutoff (see “Results”). The most variable splicing events were included in a patient-PSI matrix (Supplementary Data 1) containing 500–600 events depending upon the AS type. The patient-PSI matrix was used as an input for consensus clustering based on the non-negative matrix factorization (NMF) method^35^ as implemented in the NMF CRAN package (version 0.21.0; https://cran.r-project.org/web/packages/NMF/vignettes/NMF-vignette.pdf). NMF clustering was run using the default parameters, unless specified otherwise (see https://github.com/danjst/PDAC_2020). Euclidean distance was used as the distance metric and the cluster number was selected based on the cophenetic correlation coefficient values as an indicator of cluster stability. Clusters were evaluated based on the following validation metrics: RMSSTD, *r*-squared, and the SD validity index^37^. Top NMF events were found using the extractFeatures function in the NMF package that selects the top contributing features for each cluster based on the method defined by Kim et al.^96^ To measure the similarity between different cluster types, AMI^97–99^ and adjusted Rand index scores^100^ were used.

### Hierarchical and *k*-means clustering based on IR events

Hierarchical and *k*-means clustering was performed using the ConsensusClusterPlus (https://www.bioconductor.org/packages/release/bioc/html/ConsensusClusterPlus.html)^101^ Bioconductor package^102^. The similarity of the clusters was measured using the same methods as described for NMF clustering.

### Differential splicing analysis for IR events

Differential splicing analysis was conducted via Spladder^34^ (based on a generalized linear model) using default parameters (confidence level of 3 (highest) and using the merge-graphs strategy as described in https://spladder.readthedocs.io/en/latest/spladder_modes.html#the-test-mode). First, all the significant IR events with adjusted *p* < 0.05 (Benjamini−Hochberg procedure) were selected from the Spladder output. Significant events containing ≥10% NA values in the patient-PSI matrix were removed. Events were filtered further to remove events with an absolute difference in percent NA values between cluster 1 and 2 of ≥5%. Finally, the events with |ΔPSI_mean_| > 0.1 were selected, where ΔPSI_mean_ is the difference between mean PSIs for an event in IR clusters 1 and 2. This resulted in 262 differential IR events (Supplementary Data 5). Intron locations were determined by comparing intronic coordinates with coding region and UTR region coordinates of genes retrieved from the UCSC Data Browser (https://genome.ucsc.edu/cgi-bin/hgTables)^103^. Changes in intron retention were visualized with the Integrated Genomics Viewer^104^.

### Differential gene expression analysis

Differential gene expression between clusters was conducted using DESeq2 package (version 1.20.0; https://bioconductor.org/packages/release/bioc/html/DESeq2.html)^105^ based on raw RNA-seq counts acquired from GDC. Default DESeq2 parameters were used. The Wald test was used to calculate *p* values and log_2_fold changes were derived from maximum likelihood estimation (MLE) of IR-1_counts/IR-2_counts. Only the significant (adjusted *p* < 0.05; Benjamini−Hochberg procedure) gene expression changes were selected. Heatmaps were generated using the pheatmap (version 1.0.12) R package^106^. Numerical input was *z*-score normalized prior to heatmap plotting. Network analysis was performed using STRINGapp (http://apps.cytoscape.org/apps/stringapp)^107^ on Cytoscape^108^.

### Survival analysis of AS clusters

Clinical outcomes based on PFI, OS, and DSS for AS-based clusters were determined using the log-rank (Mantel−Cox) test. Clinical data were obtained from Liu et al.^41^ Clinical outcomes between clusters were considered significantly different at *p* < 0.05. Prism software (GraphPad Software, San Diego, CA) was used for statistical tests and plotting data. Multivariate Cox regression analysis based on the Cox Proportional-Hazards Model was conducted using the R “survival” package (version 2.44, https://cran.r-project.org/web/packages/survival/index.html)^109,110^. Covariates included in the analysis were cluster, age, sex, and history of pancreatitis. A *p* value of < 0.05 was used as a cutoff for significance.

Analysis of prognostic IR events and RBPs was performed using Survminer^111^ (version 0.4.4, https://cran.r-project.org/web/packages/survminer/index.html) R package. Survminer functions surv_cutpoint() and surv_categorize() were used to determine optimal PSI and expression cutpoints to categorize PDAC patients into PSI- and expression-based groups (“high” and “low”). Significance (*p* values) of comparisons were determined using log-rank (Mantel−Cox) test. The *p* values were adjusted for multiple comparison correction (Benjamini−Hochberg procedure) to determine the final list of predictive events and RBPs (Supplementary Data 7 and 14, respectively).

### Gene ontology analysis

Gene ontology (GO) analysis of a gene list was performed using the g:profiler program^112^ (https://biit.cs.ut.ee/gprofiler/gost) as described^113^. For statistical testing, all annotated genes were used as background. For multiple testing correction, the g:SCS algorithm was used (default method). Only GO terms related to molecular function and biological process were considered for the analysis, unless specified otherwise.

### Oncogene and tumor suppressor identification

Genes were identified as oncogenes, tumor suppressors or “both”, based on the combined datasets of the COSMIC Cancer Gene Census (https://cancer.sanger.ac.uk/census)^114^ and the OncoKB Cancer Gene List (https://oncokb.org/cancerGenes)^115^. In the few instances in which one gene was classified as an “oncogene” in one list, but a “tumor suppressor” in the other, we classified the gene as “both”.

### Motif analysis

Motif analysis for the set of 262 differentially retained introns between PDAC IR clusters was conducted using the Analysis of Motif Enrichment (AME) program^116^ of the MEME suite (meme-suite.org/tools/ame)^117^. The 4852 introns whose retention levels did not change significantly (|ΔPSI_mean_| < 0.1) between the IR clusters were used as “control sequences.” The input RNA motifs were from the “Ray2013 Homo Sapiens” database^118^. The sequence scoring method and motif enrichment tests used were average odds score and Fisher’s exact test, respectively.

### Transcription factor analysis

Transcription factor analysis was conducted using the web-based ChEA3 (ChIP-X Enrichment Analysis, v3) program (amp.pharm.mssm.edu/chea3/)^61^. Separate analyses were conducted for the lists of RBPs that were significantly upregulated or downregulated in IR-1. Top transcription factors were identified based on the number of RBPs (from the input list) that were previously reported as targets.

### IR-based clustering of other cancer types

The Spladder output files (patient-PSI matrix) for IR events for each of 16 cancer types were obtained from Kahles et al. (gdc.cancer.gov/about-data/publications/PanCanAtlas-Splicing-2018)^31^. Only tumors with ≥70% purity (based on consensus purity estimate scores^119^) were included. NMF-based unsupervised clustering was conducted on the most variable events (s.d. > 0.1) as described in the above section. Clinical outcome data (OS, PFI, and DSS) for the patients in the IR clusters were analyzed with the log-rank (Mantel−Cox) test using the Survival^109,110^ and Survminer R packages^111^. Cancer types showing significant clinical outcome differences (adjusted *p* < 0.05, Benjamini−Hochberg procedure) between the IR clusters were considered for further analysis.

### Correlation analysis

For the PDAC IR clusters, pairwise correlation analysis between expression counts of 258 differentially expressed RBPs and PSI values for 262 differentially spliced IR events was conducted via the cor.test function from the stats R package^120^. RBP-IR event correlation was determined to be significant if the Pearson correlation value was ≥0.7 and the adjusted *p* value was < 0.05 (Benjamini−Hochberg procedure).

### Reporting summary

Further information on research design is available in the Nature Research Reporting Summary linked to this article.

## Supplementary information

Supplementary Information

Supplementary Data 1

Supplementary Data 2

Supplementary Data 3

Supplementary Data 4

Supplementary Data 5

Supplementary Data 6

Supplementary Data 7

Supplementary Data 8

Supplementary Data 9

Supplementary Data 10

Supplementary Data 11

Supplementary Data 12

Supplementary Data 13

Supplementary Data 14

Supplementary Data 15

Supplementary Data 16

Supplementary Data 17

Supplementary Data 18

Reporting Summary Checklist

## Data Availability

RNA-seq count files for TCGA-PAAD, TCGA-PRAD, and TCGA-KIRC patient cohorts used in this work are publicly available for download from the NCI Genomic Data Commons^121^ (https://gdc.cancer.gov/). Controlled access bam files for TCGA-PAAD are available via dbGap with an approved protocol. CNV and mutation data for the patients were acquired from the publicly accessible cBioPortal for Cancer Genomics database^122^ (https://www.cbioportal.org/). The Spladder output files for 16 different cancer types are publicly available from GDC (gdc.cancer.gov/about-data/publications/PanCanAtlas-Splicing-2018). All clinical outcome data for patients were obtained from the Supplementary information that is part of the open access work (Liu et al.^41^), published by the TCGA network.

## Code availability

All of the software packages used in this study are listed in Supplementary Data 18. More details about the steps are presented here: https://github.com/danjst/PDAC_2020.
