# Supplementary material for: Intron retention is a robust marker of intertumoral heterogeneity in pancreatic ductal adenocarcinoma
Source: NPJ Genom Med. 2020 Dec 11;5:55. doi: 10.1038/s41525-020-00159-4 (PMC7733475; doi:10.1038/s41525-020-00159-4)
Supplement: Supplementary file 1 — Supplementary Information [file 41525_2020_159_MOESM1_ESM.pdf]

Intron retention is a robust marker of intertumoral heterogeneity in pancreatic ductal adenocarcinoma

Daniel J. Tan, Mithun Mitra, Alec M. Chiu, and Hilary A. Coller

Supplementary Information

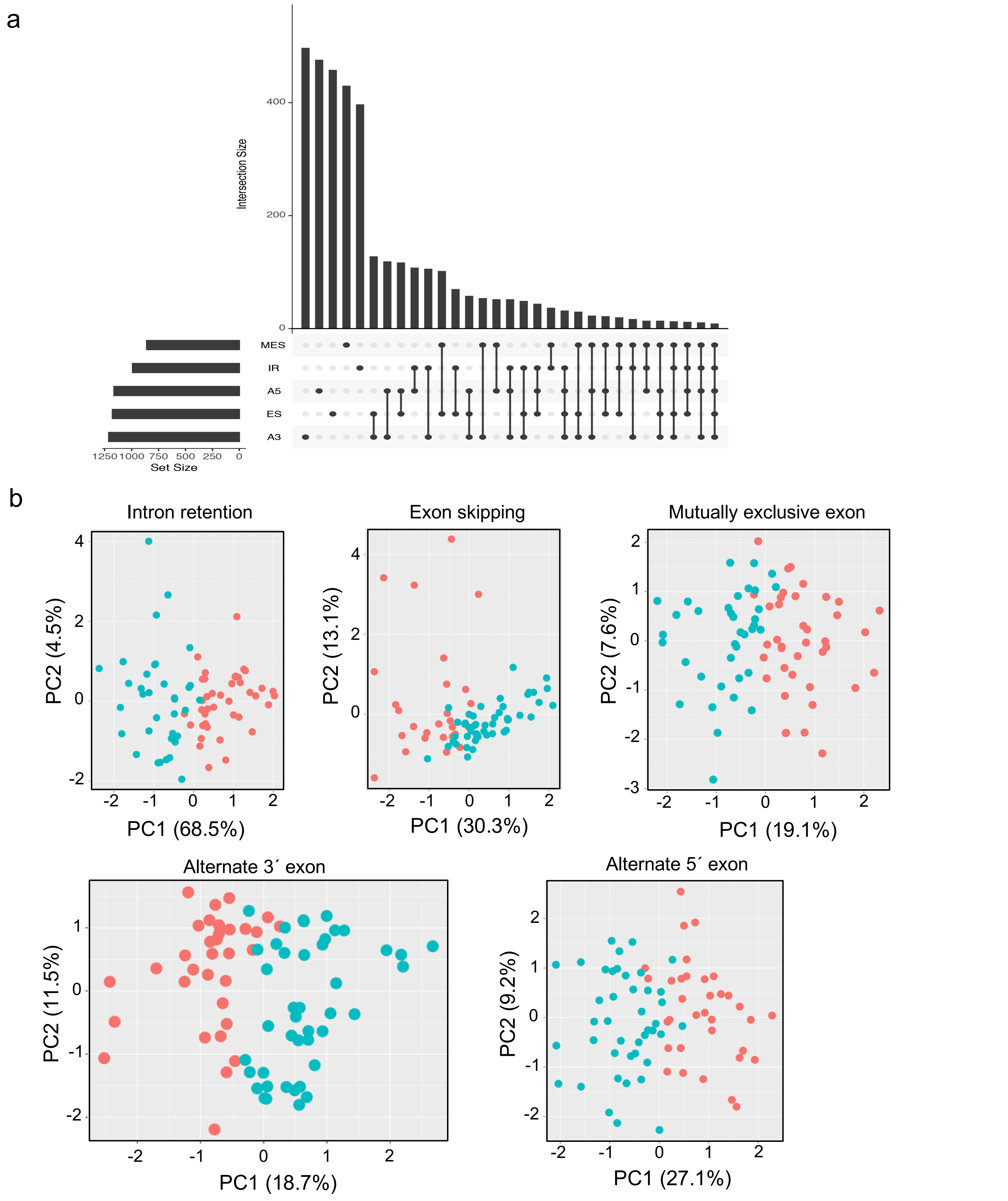

Supplementary Figure 1: Overlap among genes based on AS types and PCA plots for different AS-based clusters. (**a**) Upset plot showing the overlap among the genes corresponding to the most variable

events for each of the five AS types (MES, IR, A5, ES, and A3). These variable AS events were used as inputs for NMF clustering. The size of the set composed of splicing events for each of the five AS types is plotted on the left. (**b**) PCA plots showing the two clusters (indicated by cyan and red filled circles) for each of the five AS types. Only the first two principal components (PCs) are shown. The number in parenthesis indicates the variance explained by that PC.

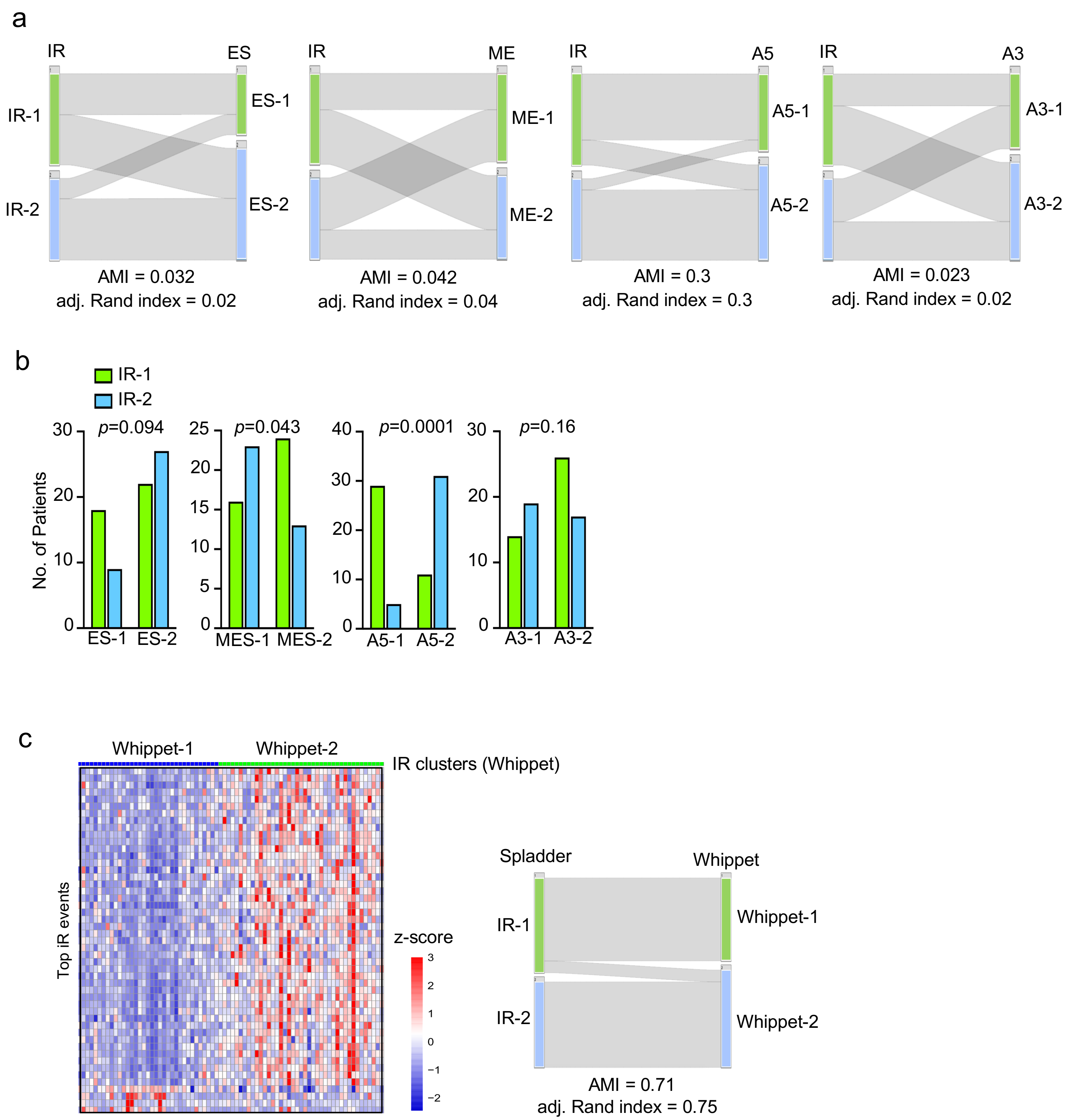

Supplementary Figure 2: Similarity of IR-based clusters to A5-based clusters and Whippet-derived IR clusters. (**a**) StratomeX plots comparing the clusters based on IR with clusters generated from ES, ME, A5, and A3 data. The values for AMI and adjusted Rand Index are also shown. (**b**) Bar plots showing the classification of ES, ME, A5, and A3 clusters based on IR clusters. *p* values were obtained from two-tailed Fisher's exact test. (**c**) Heatmap (left panel) showing the variation in IR levels of top NMF

events in the two Whippet-based IR clusters. PSI values for the events (rows) were converted to z-scores. Red and blue colors indicate high and low IR levels, respectively. A StratomeX plot (right panel) shows the comparison between Spladder- and Whippet-based IR clusters. The AMI and adjusted Rand index metrics for comparison are also shown.

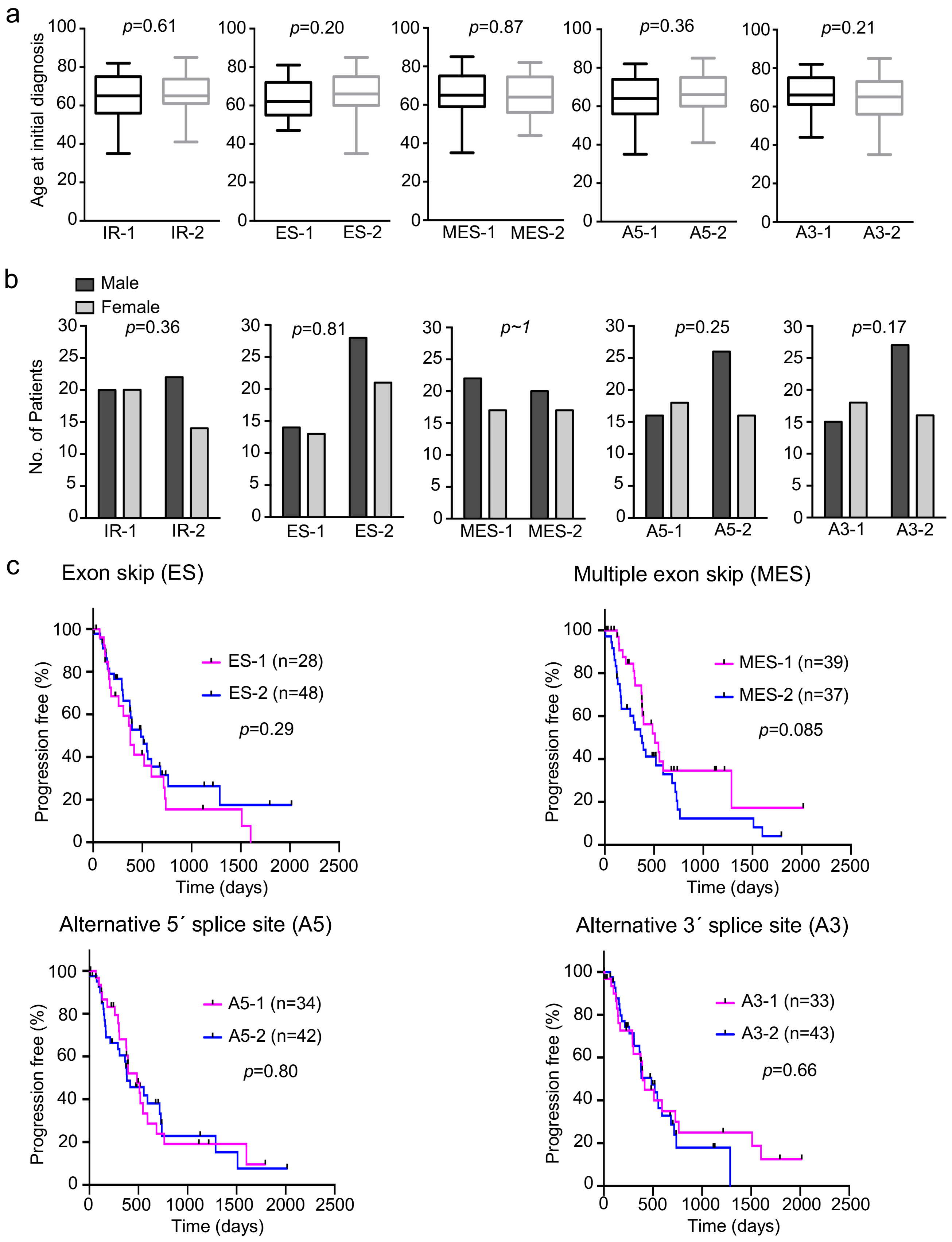

Supplementary Figure 3: AS clusters do not correlate with demographic data, and clusters based on different AS types (except IR) do not differ in PFI outcomes. (**a, b**) Comparison between the two patient

clusters for each of the five AS types on the basis of age at initial diagnosis (**a**) and gender (**b**). Box plots in **a** show the 25th and 75th percentiles, median, and whiskers that extend to the minimum and maximum values. (**c**) PFI plots for clusters based on ES, MES, A5, and A3 events, respectively. *p* values were determined by log rank (Mantel-Cox) test.

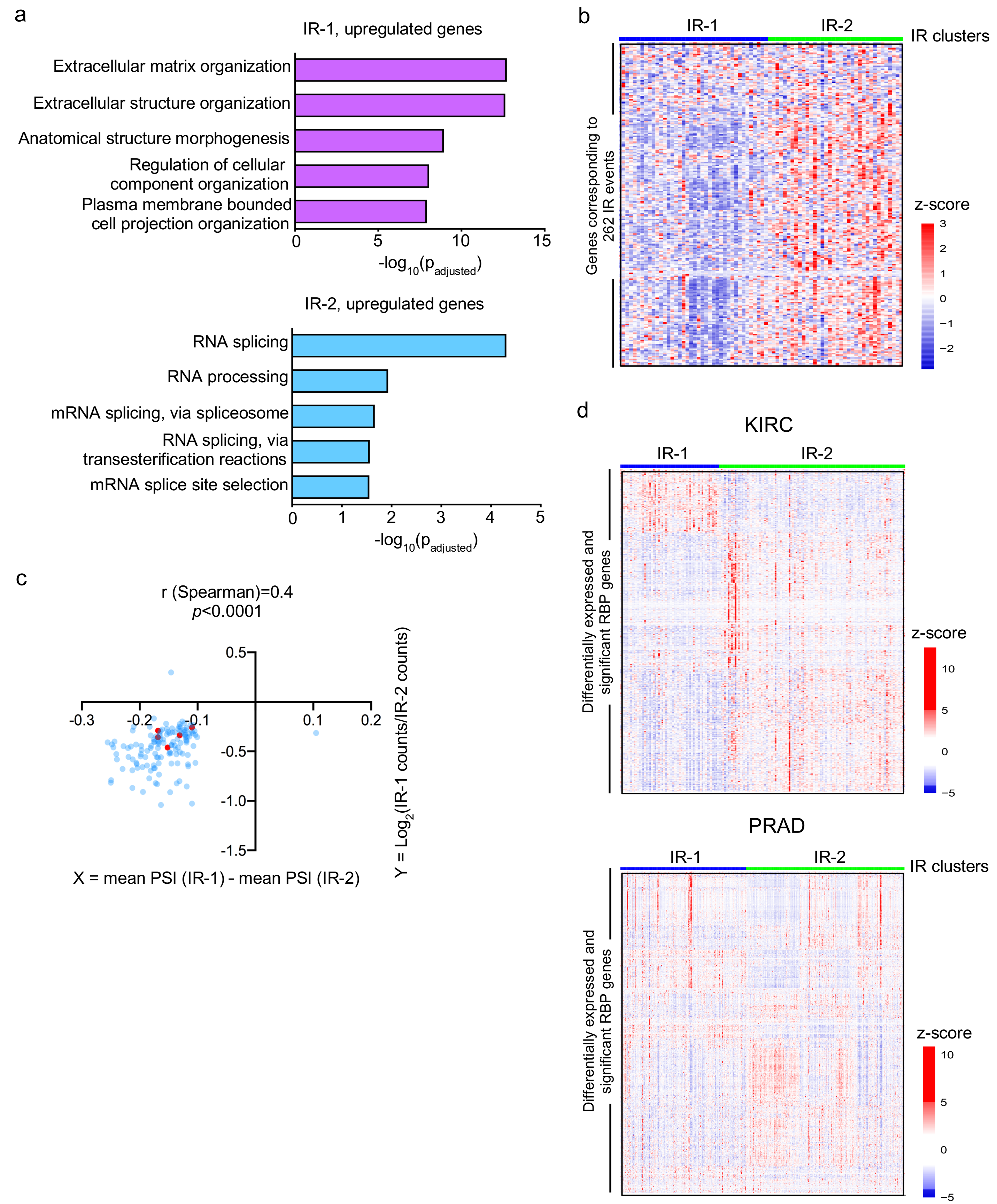

Supplementary Figure 4: Analysis of GO terms and differential gene expression patterns for IR-based clusters. (**a**) GO analysis of the genes that were significantly upregulated (top panel) in IR-1 or

upregulated in IR-2 (bottom panel) based on differential gene expression analysis between the two IR clusters. (**b**) A heatmap displaying the change in gene expression (log_2Counts) across the two IR clusters for genes corresponding to the 262 differential IR events is shown. The red and blue colors (z-score scale) indicate high and low gene expression levels, respectively. (**c**) Correlation analysis between the mean PSI difference between the two IR clusters vs. the log_2fold change in gene expression between the IR clusters for the genes corresponding to those IR events. Only IR events in the 262 differentially retained intron set for which the corresponding genes significantly change in expression between the two IR clusters were used. If a gene was related to more than one IR event, only the event with maximum mean PSI difference was used. The five IR events related to oncogenes are shown in red. (**d**) Heatmaps showing the gene expression pattern (log_2counts) for RBP genes that change significantly in gene expression (from DESeq2 analysis) between the two IR clusters for KIRC (top) and PRAD (bottom), respectively.

Supplementary Data

Supplementary Data 1: Patient-PSI matrix for the most variable events for the five AS types.

Supplementary Data 2: Cluster assignments and list of top NMF events.

Supplementary Data 3: Clustering metrics for the AS-based clusters.

Supplementary Data 4: Comparison between different AS- and gene expression-based patient clusters and clinical outcome for non-IR and gene expression-based clusters.

Supplementary Data 5: List of differentially retained introns between IR clusters.

Supplementary Data 6: GO analysis of the genes corresponding to 262 differentially retained introns between IR clusters.

Supplementary Data 7: List of significant predictive IR events.

Supplementary Data 8: Mutations and CNAs associated with IR clusters.

Supplementary Data 9: Mutations and CNAs associated with genes corresponding to 262 IR events.

Supplementary Data 10: Differentially expressed genes between the IR clusters.

Supplementary Data 11: Significant motifs and associated RBPs for 262 retained introns.

Supplementary Data 12: Correlation between RBP expression and intron retention levels.

Supplementary Data 13: Transcription factors regulating RBPs.

Supplementary Data 14: List of significant predictive RBP genes.

Supplementary Data 15: Top IR events for different cancer types.

Supplementary Data 16: Differential gene expression analysis between IR clusters for KIRC and PRAD.

Supplementary Data 17: Comparison of significant RBPs between PDAC, KIRC, and PRAD.

Supplementary Data 18: List of software packages used for this study.
