# Supplementary material for: Intron retention is a robust marker of intertumoral heterogeneity in pancreatic ductal adenocarcinoma
Source: NPJ Genom Med. 2020 Dec 11;5:55. doi: 10.1038/s41525-020-00159-4 (PMC7733475; doi:10.1038/s41525-020-00159-4)
Supplement: Supplementary file 20 — Reporting Summary Checklist [file 41525_2020_159_MOESM20_ESM.pdf]

nature research

Corresponding author(s): Hilary Coller

Reporting Summary

Nature Research wishes to improve the reproducibility of the work that we publish. This form provides structure for consistency and transparency in reporting. For further information on Nature Research policies, see Authors & Referees and the Editorial Policy Checklist.

Statistical parameters

When statistical analyses are reported, confirm that the following items are present in the relevant location (e.g. figure legend, table legend, main text, or Methods section).

n/a	Confirmed	
☐	☒	The exact sample size (n) for each experimental group/condition, given as a discrete number and unit of measurement
☐	☒	An indication of whether measurements were taken from distinct samples or whether the same sample was measured repeatedly
☐	☒	The statistical test(s) used AND whether they are one- or two-sided<br>*Only common tests should be described solely by name; describe more complex techniques in the Methods section.*
☒	☐	A description of all covariates tested
☐	☒	A description of any assumptions or corrections, such as tests of normality and adjustment for multiple comparisons
☒	☐	A full description of the statistics including central tendency (e.g. means) or other basic estimates (e.g. regression coefficient) AND variation (e.g. standard deviation) or associated estimates of uncertainty (e.g. confidence intervals)
☐	☒	For null hypothesis testing, the test statistic (e.g. F, t, r) with confidence intervals, effect sizes, degrees of freedom and P value noted<br>*Give P values as exact values whenever suitable.*
☒	☐	For Bayesian analysis, information on the choice of priors and Markov chain Monte Carlo settings
☐	☒	For hierarchical and complex designs, identification of the appropriate level for tests and full reporting of outcomes
☐	☒	Estimates of effect sizes (e.g. Cohen's d, Pearson's r), indicating how they were calculated
☐	☒	Clearly defined error bars<br>*State explicitly what error bars represent (e.g. SD, SE, CI)*

Our web collection on statistics for biologists may be useful.

Software and code

Policy information about availability of computer code

Data collection	All sequencing-related data used in this work are publicly available from GDC (https://portal.gdc.cancer.gov/). CNV and mutation data for the patients were acquired from the cBioPortal for Cancer Genomics database (https://www.cbioportal.org/). All survival and clinical data for patients were obtained from Liu et al. and the Cancer Genome Atlas, respectively.
Data analysis	All of the software packages used in this study are listed in Supplementary Table 18. More details about the steps are presented here: https://github.com/danjst/PDAC_2020.

For manuscripts utilizing custom algorithms or software that are central to the research but not yet described in published literature, software must be made available to editors/reviewers upon request. We strongly encourage code deposition in a community repository (e.g. GitHub). See the Nature Research guidelines for submitting code & software for further information.

Data

Policy information about availability of data

All manuscripts must include a data availability statement. This statement should provide the following information, where applicable:

- Accession codes, unique identifiers, or web links for publicly available datasets
- A list of figures that have associated raw data
- A description of any restrictions on data availability

All sequencing-related data used in this work are publicly available from GDC (https://portal.gdc.cancer.gov/). CNV and mutation data for the patients were acquired from the cBioPortal for Cancer Genomics database (https://www.cbioportal.org/). All survival and clinical data for patients were obtained from Liu et al. and the Cancer Genome Atlas, respectively.

Field-specific reporting

Please select the best fit for your research. If you are not sure, read the appropriate sections before making your selection.

☒ Life sciences ☐ Behavioural & social sciences ☐ Ecological, evolutionary & environmental sciences

For a reference copy of the document with all sections, see nature.com/authors/policies/ReportingSummary-flat.pdf

Life sciences study design

All studies must disclose on these points even when the disclosure is negative.

Sample size	76 high-purity PDAC samples
Data exclusions	For the initial analysis, purity was >= 33%
Replication	findings were replicated in a set of 73 low-purity PDAC samples
Randomization	N/A, no randomization
Blinding	N/A, no measurements were taken

Reporting for specific materials, systems and methods

Materials & experimental systems

n/a	Involved in the study	
☒	☐	Unique biological materials
☒	☐	Antibodies
☒	☐	Eukaryotic cell lines
☒	☐	Palaeontology
☒	☐	Animals and other organisms
☒	☐	Human research participants

Methods

n/a	Involved in the study	
☒	☐	ChIP-seq
☒	☐	Flow cytometry
☒	☐	MRI-based neuroimaging
